# Synthesis, molecular docking, ctDNA interaction, DFT calculation and evaluation of antiproliferative and anti-*Toxoplasma gondii* activities of 2,4-diaminotriazine-thiazole derivatives

**DOI:** 10.1007/s00044-018-2136-6

**Published:** 2018-02-06

**Authors:** Krzysztof Z. Łączkowski, Joanna Anusiak, Marta Świtalska, Katarzyna Dzitko, Joanna Cytarska, Angelika Baranowska-Łączkowska, Tomasz Plech, Agata Paneth, Joanna Wietrzyk, Joanna Białczyk

**Affiliations:** 1 Department of Chemical Technology and Pharmaceuticals, Faculty of Pharmacy, Collegium Medicum, Nicolaus Copernicus University, Jurasza 2, 85-089 Bydgoszcz, Poland; 2 Institute of Immunology and Experimental Therapy, Polish Academy of Sciences, Rudolfa Weigla 12, 53-114 Wrocław, Poland; 3 Department of Immunoparasitology, University of Lodz, Banacha 12/16, 90-237 Lodz, Poland; 4 Institute of Physics, Kazimierz Wielki University, Plac Weyssenhoffa 11, 85-072 Bydgoszcz, Poland; 5 Department of Pharmacology, Faculty of Health Sciences, Medical University of Lublin, Chodźki 4a, 20-093 Lublin, Poland; 6 Department of Organic Chemistry, Faculty of Pharmacy, Medical University of Lublin, Chodźki 4a, 20-093 Lublin, Poland

**Keywords:** Antiproliferative activity, *Toxoplasma gondii*, Triazine, Thiazole, Topoisomerase, DNA

## Abstract

Synthesis, characterization, and investigation of antiproliferative activities against human cancer cell lines (MV4-11, MCF-7, and A549) and *Toxoplasma gondii* parasite of twelve novel 2,4-diaminotriazine-thiazoles are presented. The toxicity of the compounds was studied at three different cell types, normal mouse fibroblast (Balb/3T3), mouse fibroblast (L929), and human VERO cells. The structures of novel compounds were determined using ^1^H and ^13^C NMR, FAB(+)-MS, and elemental analyses. Among the derivatives, **4a**–**k** showed very high activity against MV4-11 cell line with IC_50_ values between 1.13 and 3.21 µg/ml. Additionally, the cytotoxic activity of compounds **4a**–**k** against normal mouse fibroblast Balb/3T3 cells is about 20–100 times lower than against cancer cell lines. According to our results, compounds **4a**, **4b**, **4d**, and **4i** have very strong activity against human breast carcinoma MCF-7, with IC_50_ values from 3.18 to 4.28 µg/ml. Moreover, diaminotriazines **4a**–**l** showed significant anti-*Toxoplasma gondii* activity, with IC_50_ values 9–68 times lower than those observed for sulfadiazine. Molecular docking studies indicated DNA-binding site of hTopoI and hTopoII as possible anticancer targets and purine nucleoside phosphorylase as possible *anti*-toxoplasmosis target. Our UV–Vis spectroscopic results indicate also that diaminotriazine-thiazoles tends to interact with DNA by intercalation. Additionally, the structure and the interaction and binding energies of a model complex formed by compound **4a** and two thymine molecules are investigated using quantum mechanical methods.

## Introduction

The annual incidence of cancer is increasing, making cancer the second leading reason of death in Western countries after heart disease (Boyle and Levin 2008). The WHO report estimates the number of new cancer cases in 2015 being in the order of 14.1 million, and the number of cancer-related deaths—8.8 million. In 2016 the most common cancers were lung, breast, and prostate cancer. The number of new diagnoses is estimated to reach 19.3 million by 2025 (Ferlay et al. 2013).

Another global problem is toxoplasmosis, due to the extremely high seroprevalence found both in humans and livestock range from less than 10 to over 90% (Robert-Gangneux and Dardé 2012). Toxoplasmosis is a disease caused by a cosmopolitan protozoan of the *Toxoplasma* genus, comprising only one species *Toxoplasma gondii*, in which, as in the other members of the *Apicomplexa* subtype, one can distinguish two phases of the life cycle. The first, sexual, is called sporogony and occurs in gastrointestinal tract of all felines (the only known definitive hosts). The second one, asexual-schizogony occurs in the organisms of various birds, and mammals including humans (intermediate hosts) (Johnson 1998). *Toxoplasma gondii* is an obligatory intracellular parasite actively and rapidly penetrating all nucleated cells, which favors the colonization of the infected host. Many infected individuals show no symptoms, although in some cases flu symptoms may occur (e.g., high temperature and muscle aches). However, in the case of immunocompromised individuals with Hodgkin’s disease, myeloma, melanoma, leukemia, and AIDS, toxoplasmosis is reported to increase mortality (Robert-Gangneux and Dardé 2012; Contini 2008; Israelski and Remington 1993; Basavaraju 2016).

An overview of current chemotherapy methods in cancer and toxoplasmosis indicates that commonly used medications are not satisfactory. The anticancer drugs damage also healthy cells, while those used in toxoplasmosis treatment do not eradicate parasite cysts from the infected host organism, and display numerous and serious side effects (Widakowich et al. 2007; Alday and Doggett 2017). Therefore, the search for less toxic compounds, characterized by high selectivity, is crucial in the fight against cancer and toxoplasmosis. 2,4-Diaminotriazine derivatives exhibit diverse biological activities, such as potentially *Mycobacterium tuberculosis* DHFR inhibitors (Lele et al. 2015), antimalarial (Agarwal et al. 2005), anti-HIV (Patel et al. 2012), and anticancer agents (Sączewski et al. 2006; Sączewski and Bułakowska 2006). Some triazines have already found medical use, e.g., hexamethylmelamine (Altretamine), which is used in refractory ovarian cancer (Damia and D’lncalci 1995). Triazine derivatives are also known to form highly stable complexes through hydrogen-bonding interaction with thymine–thymine or uracil–uracil (T–T, U–U) mismatch sites, thus acting as purine mimics (Yu et al. 2008; Mao and Bong 2015; Zeng et al. 2012).

Our recent research has shown that some thiazol-2-yl-hydrazine derivatives including nitrogen mustard moiety possessed high antiproliferative activity against different human cancer cells, and simultaneously were characterized by low cytotoxicity against normal mouse fibroblast Balb/3T3 (Łączkowski et al. 2014, 2016a).

Considering the unique properties of triazines and continuing our previous research on the synthesis and molecular properties of substituted thiazoles (Łączkowski et al. 2015, 2016b, c, d, e, f) we decided to design and synthesize twelve novel 2,4-diaminotriazine-thiazole derivatives and investigate their antiproliferative activity against human cancer cells lines (biphenotypic B myelomonocytic leukemia MV4-11, human breast carcinoma MCF-7, and human lung carcinoma A549) and normal mouse fibroblast (Balb/3T3) using the 3-(4,5-dimethylthiazol-2-yl)-2,5-diphenyltetrazoliun bromide (MTT) or sulforhodamine B (SRB) assays. We also investigated the intensity of *Toxoplasma gondii* BK strain intracellular proliferation in the VERO host cells. Moreover, for a comprehensive testing we also performed interaction of triazines with ctDNA using UV–Visible absorption spectroscopic technique and molecular modeling and docking studies of all compounds on the active sites of selected anticancer and anti-toxoplasmosis molecular targets. Additionally, the structure and the interaction and binding energies of a model complex formed by compound **4a** and two thymine molecules are investigated using quantum mechanical methods.

## Experimental

### Materials and methods

All experiments were carried out under air atmosphere unless stated otherwise. Reagents were generally the best quality commercial-grade products and were used without further purification. ^1^H nuclear magnetic resonance (NMR) (700 MHz) and ^13^C NMR (100 MHz) spectra were recorded on a Bruker Avance III multinuclear instrument. FAB(+)-MS was performed by the Laboratory for Analysis of Organic Compounds and Polymers of the Center for Molecular and Macromolecular Studies of the Polish Academy of Science in Łódź. MS spectra were recorded on a Finnigan MAT 95 spectrometer. Elemental analysis was performed on ELEMENTAR Vario MACRO CHN. Melting points were determined in open glass capillaries and are uncorrected. Analytical thin layer chromatography (TLC) was performed using Macherey-Nagel Polygram Sil G/UV_254_ 0.2 mm plates. 2-Chloro-4,6-diamino-1,3,5-triazine, 1-(4-aminophenyl)ethanone, acetic acid, and thiosemicarbazide, were commercial materials (Aldrich).

#### 1-(4-(4,6-Diamino-1,3,5-triazin-2-yloamino))phenyl)ethanone (**2**)

2-Chloro-4,6-diamino-1,3,5-triazine (2.92 g, 20.0 mmoles) was added to a stirred solution of 1-(4-aminophenyl)ethanone (1) (2.70 g, 20.0 mmoles) in dry dioxane (120 ml). The reaction mixture was stirred under reflux for 20 h under nitrogen atmosphere and the product was filtered off and subsequently washed with dioxane. The separated precipitate was suspended in water and neutralized with NaHCO_3_ solution and next purified on silica gel column chromatography (230–400 mesh) using (dichloromethane/methanol, 80:20, *R*_f_ = 0.72) to afford the desired product: 4.56 g, 93%; mp 230–232 °C. ^1^H NMR (DMSO-d_6_, 700 MHz): *δ* (ppm) 2.52 (s, 3H, CH_3_); 6.45 (bs, 4H, 2NH_2_); 7.84 (d, 2H, 2CH, *J* = 8.0 Hz); 7.97 (d, 2H, 2CH, *J* = 8.0 Hz); 9.33 (s, 1H, NH). ^13^C NMR (DMSO-d_6_, 100 MHz): *δ* (ppm) 26.74 (CH_3_–CO); 118.62 (2C_Ar_); 129.55 (2C_Ar_); 130.00 (C_Ar_); 146.06 (C_Ar_); 165.22 (C_triazine_); 167.62 (2C_triazine_); 196.71 (C=O). Anal. calcd. for C_11_H_12_N_6_O: C, 54.09; H, 4.95; N, 34.41. Found: C, 54.11; H, 4.90; N, 34.45.

#### (2*E*)-2-(1-(4-((4,6-diamino-1,3,5-triazin-2-yl)amino)phenyl)ethylidene)hydrazinecarbothioamide (**3**)

Thiosemicarbazide (0.30 g, 3.33 mmoles) was added to a stirred solution of 1-(4-(4,6-diamino-1,3,5-triazin-2-yloamino))phenyl)ethanone (**2**) (0.81 g, 3.33 mmoles) in 60% ethyl alcohol and then (2 ml) concentrated hydrochloric acid was added. The reaction mixture was stirred under reflux for 20 h, cooled to room temperature and separate precipitate was collected by filtration, suspended in water and neutralized with NaHCO_3_ solution. Yield: 0.77 g, 73%; mp >230 °C, (dichloromethane/methanol, 80:20, *R*_f_ = 0.44). ^1^H NMR (DMSO-d_6_, 700 MHz): *δ* (ppm) 2.28 (s, 3H, CH_3_); 6.36 (bs, 4H, 2NH_2_); 7.80 (d, 2H, 2CH, *J* = 9.0 Hz); 7.84 (d, 2H, 2CH, *J* = 9.0 Hz); 7.87 (s, 1H, NH_2_); 8.22 (s, 1H, NH_2_); 9.05 (s, 1H, NH); 10.13 (s, 1H, NH). ^13^C NMR (DMSO-d_6_, 100 MHz): *δ* (ppm) 14.23 (CH_3_–C=N); 119.16 (2C_Ar_); 127.26 (2C_Ar_); 130.52 (C_Ar_); 142.45 (C_Ar_); 148.63 (C=N); 165.22 (C_triazine_); 167.61 (2C_triazine_); 179.04 (C=S). Anal. calcd. for C_12_H_15_N_9_S: C, 45.41; H, 4.76; N, 39.72. Found: C, 45.42; H, 4.77; N, 39.76.

#### (*E*)-*N*^2^-(4-(1-(2-(4-(4-fluorophenyl)-1,3-thiazol-2-yl)hydrazinylidene)ethyl)phenyl)-1,3,5-triazine-2,4,6-triamine (**4a**). Typical Procedure

Thiosemicarbazone **3** (0.317 g, 1.0 mmoles) was added to a stirred solution of 2-bromo-1-(4-fluorophenyl)ethanone (0.217 g, 1.0 mmole) in DMF/EtOH (3: 2) (50 ml). The reaction mixture was stirred under room temperature for 2 h, and separate precipitate was collected by filtration, suspended in water and neutralized with NaHCO_3_ solution. The crude product was purified on silica gel column chromatography (230–400 mesh) using (dichloromethane/methanol, 80:20, *R*_f_ = 0.74) to afford the desired product: 0.38 g, 87%; mp 223–225 °C. ^1^H NMR (DMSO-d_6_, 700 MHz): *δ* (ppm) 2.31 (s, 3H, CH_3_); 6.39 (bs, 4H, 2NH_2_); 7.26 (m, 2H, 2CH); 7.31 (s, 1H, CH); 7.68 (d, 2H, 2CH, *J* = 9.0 Hz); 7.89 (d, 2H, 2CH, *J* = 9.0 Hz); 7.93 (m, 2H, 2CH); 9.07 (s, 1H, NH); 11.13 (s, 1H, NH). ^13^C NMR (DMSO-d_6_, 100 MHz): *δ* (ppm) 14.31 (CH_3_–C=N); 104.04 (C_thiazole_); 115.77 (C); 115.98 (C); 119.47 (2C_Ar_); 126.29 (2C_Ar_); 127.93 (2C_Ar_); 131.03 (C); 131.98 (C); 133.62 (C); 141.91 (C); 147.21 (C); 149.95 (C=N); 165.07 (C_triazine_); 167.20 (2C_triazine_); 170.57 (C_thiazole_). FAB(+)-MS (*m*/*z*, %): 436.2 [(M^+^ + 1), 100], 244.1 (70), 242.1 (80), 228.1 (100), 202.1 (40), 158.0 (20). Anal. calcd. for C_20_H_18_FN_9_S: C, 55.16; H, 4.17; N, 28.95. Found: C, 55.14; H, 4.20; N, 28.98.

#### (*E*)-*N*^2^-(4-(1-(2-(4-(4-chlorophenyl)-1,3-thiazol-2-yl)hydrazinylidene)ethyl)phenyl)-1,3,5-triazine-2,4,6-triamine (**4b**)

Yield: 0.35 g, 78%, (dichloromethane/methanol, 80:20, *R*_f_ = 0.65); mp 237–239 °C. ^1^H NMR (DMSO-d_6_, 700 MHz): *δ* (ppm) 2.31 (s, 3H, CH_3_); 6.38 (bs, 4H, 2NH_2_); 7.39 (s, 1H, CH); 7.49 (d, 2H, 2CH, *J* = 8.5 Hz); 7.68 (d, 2H, 2CH, *J* = 9.0 Hz); 7.89 (d, 2H, 2CH, *J* = 9.0 Hz); 7.91 (d, 2H, 2CH, *J* = 8.5 Hz); 9.06 (s, 1H, NH); 11.15 (bs, 1H, NH). ^13^C NMR (DMSO-d_6_, 100 MHz): *δ* (ppm) 14.32 (CH_3_–C=N); 105.11 (C_thiazole_); 119.43 (2C_Ar_); 126.29 (2C_Ar_); 127.68 (2C_Ar_); 129.08 (2C_Ar_); 130.91 (C); 132.29 (C); 134.23 (C); 142.00 (C); 147.31 (C); 149.84 (C=N); 165.16 (C_triazine_); 167.41 (2C_triazine_); 170.61 (C_thiazole_). FAB(+)-MS (*m*/*z*, %): 452.1 [(M^+^ + 1), 100], 241.1 (70), 242.1 (70), 228.1 (100), 202.1 (40), 158.0 (20), 127.0 (20). Anal. calcd. for C_20_H_18_ClN_9_S: C, 53.15; H, 4.01; N, 27.89. Found: C, 53.20; H, 4.05; N, 27.93.

#### (*E*)-*N*^2^-(4-(1-(2-(4-(4-tolyl)-1,3-thiazol-2-yl)hydrazinylidene)ethyl)phenyl)-1,3,5-triazine-2,4,6-triamine (**4c**)

Yield: 0.34 g, 79%, (dichloromethane/methanol, 80:20, *R*_f_ = 0.62); mp 231–233 °C. ^1^H NMR (DMSO-d_6_, 700 MHz): *δ* (ppm) 2.31 (s, 3H, CH_3_); 2.34 (s, 3H, CH_3_); 6.44 (bs, 4H, 2NH_2_); 7.24 (d, 2H, 2CH, *J* = 9.0 Hz); 7.24 (s, 1H, CH); 7.68 (d, 2H, 2CH, *J* = 9.0 Hz); 7.79 (d, 2H, 2CH, *J* = 9.5 Hz); 7.88 (d, 2H, 2CH, *J* = 9.5 Hz); 9.10 (s, 1H, NH); 11.10 (bs, 1H, NH). ^13^C NMR (DMSO-d_6_, 100 MHz): *δ* (ppm) 14.28 (CH_3_–C=N); 21.26 (CH_3_–Ph); 103.35 (C_thiazole_); 119.56 (2C_Ar_); 125.93 (2C_Ar_); 126.26 (2C_Ar_); 129.52 (C); 129.62 (2C_Ar_); 137.15 (C); 141.73 (C); 144.96 (C); 147.02 (C); 150.96 (C=N); 164.91 (C_triazine_); 167.76 (2C_triazine_); 170.35 (C_thiazole_). FAB(+)-MS (*m*/*z*, %): 432.1 [(M^+^ + 1), 100], 244.1 (80), 242.1 (100), 228.1 (100), 202.1 (40), 158.0 (20), 119.0 (49). Anal. calcd. for C_21_H_21_N_9_S: C, 58.45; H, 4.91; N, 29.21. Found: C, 58.41; H, 4.88; N, 29.24.

#### (*E*)-*N*^2^-(4-(1-(2-(4-(4-bromophenyl)-1,3-thiazol-2-yl)hydrazinylidene)ethyl)phenyl)-1,3,5-triazine-2,4,6-triamine (**4d**)

Yield: 0.23 g, 46%, (dichloromethane/methanol, 80:20, *R*_f_ = 0.51); mp 226–230 °C. ^1^H NMR (DMSO-d_6_, 700 MHz): *δ* (ppm) 2.31 (s, 3H, CH_3_); 6.38 (bs, 4H, 2NH_2_); 7.41 (s, 1H, CH); 7.62 (d, 2H, 2CH, *J* = 8.5 Hz); 7.68 (d, 2H, 2CH, *J* = 9.0 Hz); 7.85 (d, 2H, 2CH, *J* = 8.5 Hz); 7.89 (d, 2H, 2CH, *J* = 9.0 Hz); 9.06 (s, 1H, NH); 11.15 (bs, 1H, NH). ^13^C NMR (DMSO-d_6_, 100 MHz): *δ* (ppm) 14.32 (CH_3_–C=N); 105.23 (C_thiazole_); 119.38 (2C_Ar_); 126.29 (2C_Ar_); 128.00 (2C_Ar_); 129.53 (C); 130.88 (C); 131.99 (2C); 134.58 (C); 142.00 (C); 147.28 (C); 149.80 (C); 149.89 (C=N); 165.12 (C_triazine_); 167.34 (2C_triazine_); 170.59 (C_thiazole_). FAB(+)-MS (*m*/*z*, %): 496.1 [(M^+^ + 1), 100], 244.1 (80), 242.1 (70), 228.1 (100), 202.1 (40). Anal. calcd. for C_20_H_18_BrN_9_S: C, 48.39; H, 3.66; N, 25.40. Found: C, 48.44; H, 3.64; N, 25.42.

#### (*E*)-*N*^2^-(4-(1-(2-(4-(4-iodophenyl)-1,3-thiazol-2-yl)hydrazinylidene)ethyl)phenyl)-1,3,5-triazine-2,4,6-triamine (**4e**)

Yield: 0.49 g, 91%, (dichloromethane/methanol, 80:20, *R*_f_ = 0.79); mp 248–251 °C. ^1^H NMR (DMSO-d_6_, 700 MHz): *δ* (ppm) 2.31 (s, 3H, CH_3_); 6.50 (bs, 4H, 2NH_2_); 7.41 (s, 1H, CH); 7.68 (d, 2H, 2CH, *J* = 8.0 Hz); 7.70 (d, 2H, 2CH, *J* = 8.5 Hz); 7.79 (d, 2H, 2CH, *J* = 8.0 Hz); 7.87 (d, 2H, 2CH, *J* = 8.5 Hz); 9.15 (s, 1H, NH); 11.16 (bs, 1H, NH). ^13^C NMR (DMSO-d_6_, 100 MHz): *δ* (ppm) 14.32 (CH_3_–C=N); 93.74 (C); 105.22 (C_thiazole_); 119.57 (C); 119.77 (C); 126.31 (2C_Ar_); 128.11 (2C_Ar_); 129.52 (C); 130.82 (2C); 137.84 (2C); 138.30 (C); 141.55 (C=N); 164.60 (C_triazine_); 168.60 (2C_triazine_); 170.52 (C_thiazole_). FAB(+)-MS (*m*/*z*, %): 544.0 [(M^+^ + 1), 100], 244.1 (90), 242.1 (100), 228.1 (100), 202.1 (40), 149.0 (90). Anal. calcd. for C_20_H_18_IN_9_S: C, 44.21; H, 3.34; N, 23.20. Found: C, 44.20; H, 3.30; N, 23.24.

#### (*E*)-3-(2-(2-(1-(4-(4,6-diamino-1,3,5-triazin-2-yloamino)phenyl)ethylidene)hydrazinyl)thiazol-4-yl)-2*H*-chromen-2-on (**4f**)

Yield: 0.45 g, 93%, (dichloromethane/methanol, 80:20, *R*_f_ = 0.80); mp 285–289 °C. ^1^H NMR (DMSO-d_6_, 700 MHz): *δ* (ppm) 2.32 (s, 3H, CH_3_); 6.40 (bs, 4H, 2NH_2_); 7.42 (t, 1H, CH, *J* = 7.5 Hz); 7.49 (d, 1H, CH, *J* = 8.0 Hz); 7.66 (t, 1H, CH, *J* = 8.0 Hz); 7.69 (d, 2H, 2CH, *J* = 9.0 Hz); 7.81 (s, 1H, CH); 7.85 (d, 1H, CH, *J* = 8.5 Hz); 7.89 (d, 2H, 2CH, *J* = 9.0 Hz); 8.61 (s, 1H, CH); 9.09 (s, 1H, NH); 11.22 (s, 1H, NH). ^13^C NMR (DMSO-d_6_, 100 MHz): *δ* (ppm) 14.34 (CH_3_–C=N); 111.38 (C_thiazole_); 116.36 (C); 119.60 (2C_Ar_); 121.19 (C); 125.21 (C); 126.33 (2C_Ar_); 129.19 (C); 129.52 (C); 131.15 (C); 132.12 (C); 138.45 (C); 141.71 (C); 144.47 (C); 147.50 (C); 152.76 (C); 159.24 (C); 164.74 (C_triazine_); 166.25 (2C_triazine_); 169.90 (C_thiazole_). FAB(+)-MS (*m*/*z*, %): 486.2 [(M^+^ + 1), 100], 244.1 (100), 243.1 (80), 228.1 (80), 202.1 (30), 185.1 (100), 149.1 (70). Anal. calcd. for C_23_H_19_N_9_O_2_S: C, 56.90; H, 3.94; N, 25.96. Found: C, 56.94; H, 3.90; N, 26.01.

#### (*E*)-*N*^*2*^-(4-(2-(2-(1-(4-(4,6-diamino-1,3,5-triazin-2-yloamino)phenyl)ethylidene)hydraziny)thiazol-4-yl)phenyl)-acetamide (**4g**)

Yield: 0.28 g, 59%, (dichloromethane/methanol, 80:20, *R*_f_ = 0.57); mp >300 °C. ^1^H NMR (DMSO-d_6_, 700 MHz): *δ* (ppm) 2.08 (s, 3H, CH_3_); 2.30 (s, 3H, CH_3_); 6.36 (bs, 4H, 2NH_2_); 7.20 (s, 1H, CH); 7.64 (d, 2H, 2CH, *J* = 9.0 Hz); 7.67 (d, 2H, 2CH, *J* = 8.5 Hz); 7.81 (d, 2H, 2CH, *J* = 9.0 Hz); 7.88 (d, 2H, 2CH, *J* = 8.5 Hz); 9.04 (s, 1H, NH); 10.02 (s, 1H, NH); 11.10 (s, 1H, NH). ^13^C NMR (DMSO-d_6_, 100 MHz): *δ* (ppm) 14.29 (CH_3_–C=N); 24.52 (CH_3_–CONH); 102.93 (C_thiazole_); 119.33 (2C_Ar_); 119.38 (2C_Ar_); 126.25 (2C_Ar_); 126.37 (2C_Ar_); 129.54 (C); 130.91 (C); 139.11 (C); 141.99 (C); 150.43 (C=N); 165.19 (C); 167.54 (2C_triazine_); 168.74 (C); 170.36 (C_thiazole_), 196.90 (C=O). FAB(+)-MS (*m*/*z*, %): 475.2 [(M^+^ + 1), 100], 312.4 (90), 284.3 (40), 244.2 (100), 242.2 (70), 228.1 (70), 149.0 (40). Anal. calcd. for C_22_H_22_N_10_OS: C, 56.68; H, 4.67; N, 29.52. Found: C, 56.67; H, 4.70; N, 29.55.

#### (*E*)-*N*^2^-(4-(1-(2-(4-(4-nitrophenyl)-1,3-thiazol-2-yl)hydrazinylidene)ethyl)phenyl)-1,3,5-triazine-2,4,6-triamine (**4h**)

Yield: 0.39 g, 85%, (dichloromethane/methanol, 80:20, *R*_f_ = 0.76); mp 269–271 °C. ^1^H NMR (DMSO-d_6_, 700 MHz): *δ* (ppm) 2.32 (s, 3H, CH_3_); 6.37 (bs, 4H, 2NH_2_); 7.68 (d, 2H, 2CH, *J* = 9.0 Hz); 7.74 (s, 1H, CH); 7.89 (d, 2H, 2CH, *J* = 9.0 Hz); 8.16 (d, 2H, 2CH, *J* = 9.0 Hz); 8.31 (d, 2H, 2CH, *J* = 9.0 Hz); 9.07 (s, 1H, NH); 11.28 (s, 1H, NH). ^13^C NMR (DMSO-d_6_, 100 MHz): *δ* (ppm) 14.38 (CH_3_–C=N); 109.25 (C_thiazole_); 119.38 (2C_Ar_); 124.58 (2C_Ar_); 126.35 (2C_Ar_); 126.75 (2C_Ar_); 129.53 (C); 130.78 (C); 141.36 (C); 146.59 (C); 147.67 (C); 149.06 (C=N); 165.17 (C_triazine_); 167.45 (2C_triazine_); 170.88 (C_thiazole_). FAB(+)-MS (*m*/*z*, %): 463.2 [(M^+^ + 1), 100], 312.4 (60), 244.2 (90), 242.2 (90), 228.1 (100), 202.1 (40), 185.1 (80), 149.0 (60), 147.0 (90), 109.1 (50). Anal. calcd. for C_20_H_18_N_10_O_2_S: C, 51.94; H, 3.92; N, 30.29. Found: C, 51.97; H, 3.90; N, 30.33.

#### (*E*)-*N*^2^-(4-(1-(2-(4-(3,4-dichlorophenyl)-1,3-thiazol-2-yl)hydrazinylidene)ethyl)phenyl)-1,3,5-triazine-2,4,6-triamine (**4i**)

Yield: 0.36 g, 74%, (dichloromethane/methanol, 80:20, *R*_f_ = 0.72); mp 214–216 °C. ^1^H NMR (DMSO-d_6_, 700 MHz): *δ* (ppm) 2.31 (s, 3H, CH_3_); 6.37 (bs, 4H, 2NH_2_); 7.56 (s, 1H, CH); 7.64–7.71 (m, 3H, 3CH); 7.81–7.91 (m, 3H, 3CH); 8.14 (d, 2H, 2CH, *J* = 2.0 Hz); 9.06 (s, 1H, NH); 11.18 (s, 1H, NH). ^13^C NMR (DMSO-d_6_, 100 MHz): *δ* (ppm) 14.33 (CH_3_–C=N); 106.58 (C_thiazole_); 119.54 (2C_Ar_); 125.97 (C); 126.32 (2C_Ar_); 127.66 (C); 130.04 (C); 130.81 (C); 131.32 (C); 131.90 (C); 135.92 (C); 142.07 (C); 147.53 (C); 148.47 (C=N); 165.17 (C_triazine_); 167.47 (2C_triazine_); 170.68 (C_thiazole_). FAB(+)-MS (*m*/*z*, %): 486.2 [(M^+^ + 1), 100], 488.2 (70), 244.2 (70), 242.2 (70), 228.1 (100), 202.1 (40), 167.0 (32), 145.0 (28). Anal. calcd. For C_20_H_17_Cl_2_N_9_S: C, 49.39; H, 3.52; N, 25.92. found: C, 49.37; H, 3.49; N, 25.96.

#### (*E*)-*N*-(4-(2-(2-(1-(4-(4,6-diamino-1,3,5-triazin-2-yloamino)phenyl)ethylidene)hydrazinyl)thiazol-4-yl)phenyl)-methanesulfonamide (**4j**)

Yield: 0.35 g, 69%, (dichloromethane/methanol, 80:20, *R*_f_ = 0.60); mp 262–264 °C. ^1^H NMR (DMSO-d_6_, 700 MHz): *δ* (ppm) 2.31 (s, 3H, CH_3_); 3.04 (s, 3H, CH_3_); 6.36 (bs, 4H, 2NH_2_); 7.24 (s, 1H, CH); 7.26 (d, 2H, 2CH, *J* = 9.0 Hz); 7.67 (d, 2H, 2CH, *J* = 9.0 Hz); 7.85 (d, 2H, 2CH, *J* = 9.0 Hz); 7.88 (d, 2H, 2CH, *J* = 9.0 Hz); 9.04 (s, 1H, NH); 9.84 (s, 1H, NH); 11.12 (s, 1H, NH). ^13^C NMR (DMSO-d_6_, 100 MHz): *δ* (ppm) 14.31 (CH_3_–C=N); 26.84 (CH_3_SO_2_); 103.46 (C_thiazole_); 119.40 (2C_Ar_); 119.56 (C); 120.18 (2C_Ar_); 126.28 (2C_Ar_); 126.95 (2C_Ar_); 129.53 (C); 130.97 (C); 138.04 (C); 141.89 (C); 147.11(C=N); 165.11(C_triazine_); 167.29 (2C_triazine_); 170.45 (C_thiazole_). FAB(+)-MS (*m*/*z*, %): 511.2 [(M^+^ + 1), 100], 244.1 (100), 242.1 (100), 228.1 (100), 202.1 (40). Anal. calcd. for C_21_H_22_N_10_O_2_S_2_: C, 49.40; H, 4.34; N, 27.43. Found: C, 49.45; H, 4.35; N, 27.46.

#### (*E*)-*N*^2^-(4-(1-(2-(4-phenyl-1,3-thiazol-2-yl)hydrazinylidene)ethyl)phenyl)-1,3,5-triazine-2,4,6-triamine (**4k**)

Yield: 0.30 g, 72%, (dichloromethane/methanol, 80:20, *R*_f_ = 0.71); mp >300 °C. ^1^H NMR (DMSO-d_6_, 700 MHz): *δ* (ppm) 2.31 (s, 3H, CH_3_); 6.37 (bs, 4H, 2NH_2_); 7.32 (t, 1H, CH, *J* = 7.0 Hz); 7.33 (s, 1H, CH); 7.43 (t, 2H, 2CH, *J* = 7.0 Hz); 7.68 (d, 2H, 2CH, *J* = 9.0 Hz); 7.87–7.92 (m, 4H, 4CH); 9.05 (s, 1H, NH); 11.12 (s, 1H, NH). ^13^C NMR (DMSO-d_6_, 100 MHz): *δ* (ppm) 14.30 (CH_3_–C=N); 104.30 (C_thiazole_); 119.39 (C); 119.58 (C); 125.98 (2C_Ar_); 126.27 (C); 129.06 (2C_Ar_); 129.54 (C); 130.97 (C); 135.35 (C); 141.96 (C); 147.15 (C); 150.44 (C=N); 157.08 (C); 165.16 (C_triazine_); 167.42 (2C_triazine_); 170.47 (C_thiazole_). FAB(+)-MS (*m*/*z*, %): 418.3 [(M^+^ + 1), 100], 244.2 (90), 242.2 (80), 228.1 (100), 202.1 (40). Anal. calcd. for C_20_H_19_N_9_S: C, 57.54; H, 4.59; N, 30.19. Found: C, 57.54; H, 4.60; N, 30.21.

#### (*E*)-6-(2-(2-(1-(4-(4,6-diamino-1,3,5-triazin-2-yloamino)phenyl)ethylidene)hydrazinyl)thiazol-4-yl)-benzo[*d*]oxazol-2(3*H*)-on (**4l**)

Yield: 0.37 g, 78%, (dichloromethane/methanol, 80:20, *R*_f_ = 0.59); mp >300 °C. ^1^H NMR (DMSO-d_6_, 700 MHz): *δ* (ppm) 2.31 (s, 3H, CH_3_); 6.36 (bs, 4H, 2NH_2_); 7.14 (d, H, CH, *J* = 8.5 Hz); 7.29 (s, 1H, CH); 7.67 (d, 2H, 2CH, *J* = 8.5 Hz); 7.73 (d, 2H, 2CH, *J* = 8.5 Hz); 7.77 (s, 1H, CH); 7.88 (d, 2H, 2CH, *J* = 8.5 Hz); 9.05 (s, 1H, NH); 11.11 (s, 1H, NH); 11.73 (s, 1H, NH). ^13^C NMR (DMSO-d_6_, 100 MHz): *δ* (ppm) 14.30 (CH_3_–C=N); 107.07 (C_thiazole_); 110.17 (C); 119.34 (2C_Ar_); 119.55 (C); 121.79 (C); 126.27 (2C_Ar_); 129.52 (C); 130.16 (C); 130.88 (C); 142.02 (C); 144.22 (C); 150.39 (C=N); 154.99 (C); 165.21 (C_triazine_); 167.57 (2C_triazine_); 170.38 (C_thiazole_); 196.89 (C=O). FAB(+)-MS (*m*/*z*, %): 475.3 [(M^+^ + 1), 100], 244.2 (90), 242.2 (80), 228.1 (100), 202.1 (40), 147.0 (40). Anal. calcd. for C_21_H_18_N_19_O_2_S: C, 53.16; H, 3.82; N, 29.52. Found: C, 53.21; H, 3.84; N, 29.55.

### Antiproliferative activity

#### Cells

Biphenotypic B myelomonocytic leukemia MV4-11, human lung carcinoma A549, human breast carcinoma MCF-7, and normal mouse fibroblast BALB/3T3 cells were obtained from American Type Culture Collection (Rockville, Maryland, USA). All the cell lines are being maintained at the Institute of Immunology and Experimental Therapy, Wroclaw, Poland. MV4-11 cells were cultured in RPMI 1640 medium (Gibco, UK) with 2 mM L-glutamine adjusted to contain 1.0 mM sodium pyruvate, and 10% fetal bovine serum (FBS) (all from Sigma-Aldrich, Germany). A549 cells were cultured in RPMI 1640+ Opti-MEM (1:1) (both from Gibco, UK), MCF-7 cells in Eagle medium (IIET, Wroclaw, Poland), BALB/3T3 in Dulbecco medium (IIET, Poland) supplemented with 2 mM l-glutamine and 1.0 mM sodium pyruvate, 10% fetal bovine serum (all from Sigma-Aldrich, Germany). The MCF-7 cell culture was supplemented with 0.8 mg/l of insulin (Sigma-Aldrich, Germany). All culture media were supplemented with 100 units/ml penicillin, and 100 µg/ml streptomycin (both from Polfa Tarchomin S.A., Poland). All cell lines were grown at 37 °C with 5% CO_2_ humidified atmosphere (Łączkowski et al. 2014, 2016a).

### Compounds

Prior to usage, the compounds were dissolved in DMSO and culture medium (1: 9) to the concentration of 1 mg/ml, and subsequently diluted in culture medium to reach the required concentrations (0.1, 1, 10, and 100 µg/ml) (Łączkowski et al. 2014, 2016a).

### In vitro antiproliferative assay

Twenty-four hours prior to the addition of the tested compounds, the cells counted using Burker hemocytometer were plated in 96-well plates (Sarstedt, Germany) at a density of 1 × 10^4^ cells per well. The assay was performed after 72 h of exposure to varying concentrations of the tested agents. The in vitro cytotoxic effect of all agents was examined using the SRB assay for adherent cells (A549, MCF-7, and BALB/3T3) or MTT assay for leukemia cells (MV4-11) as described previously. The results were calculated as an inhibitory concentration 50 (IC_50_)—the concentration of tested agent which inhibits proliferation of 50% of the cancer cell population. IC values were calculated for each experiment separately and mean values ± SD are presented in the Table 1. Each compound in each concentration was tested in triplicate in a single experiment, which was repeated 3–7 times (Rubinstein et al. 1990; Bramson et al. 1995).

**Table 1:** Antiproliferative activity of thiazole-based nitrogen mustards **4a**–**l** against cancer cell lines MV4-11, MCF-7, A549, and normal mice fibroblast Balb/3T3

Triazines	R	IC_50_ (µg/ml)			
		MV4-11	MCF-7	A549	Balb/3T3
**4a**		3.21 ± 0.454	4.28 ± 0.984	38.57 ± 4.173	62.59 ± 8.686
**4b**		1.89 ± 0.45	3.23 ± 0.401	23.76 ± 4.41	>100
**4c**		3.09 ± 0.726	8.33 ± 2.83	62.37 ± 11.549	>100
**4d**		2.14 ± 0.464	3.18 ± 0.192	24.74 ± 6.099	65.16 ± 24.537
**4e**		1.13 ± 0.25	8.41 ± 0.373	45.40 ± 18.302	>100
**4f**		3.08 ± 0.851	─	─	>100
**4g**		2.76 ± 0.521	6.67 ± 2.958	─	>100
**4h**		3.01 ± 0.476	8.72 ± 5.278	33.61 ± 2.424	>100
**4i**		1.37 ± 0.186	3.69 ± 1.638	57.53 ± 20.701	>100
**4j**		1.73 ± 0.091	5.95 ± 2.165	─	>100
**4k**		2.30 ± 0.97	5.73 ± 2.054	─	>100
**4l**		>100	nt	nt	>100
*cis*-platin	─	0.22 ± 0.11	2.33 ± 0.465	2.69 ± 1.119	2.55 ± 0.588

### SRB cytotoxic test

Cells were attached to the bottom of plastic wells by fixing them with cold 50% TCA (trichloroacetic acid, POCH, Gliwice, Poland) on top of the culture medium in each well. The plates were incubated at 4 °C for 1 h and then washed five times with tap water. The cellular material fixed with TCA was stained with 0.1% sulforhodamine B (SRB, Sigma-Aldrich, Germany) and dissolved in 1% acetic acid (POCH, Gliwice, Poland) for 30 min. Unbound dye was removed by rinsing (4×) in 1% acetic acid. The protein-bound dye was extracted with 10 mM unbuffered Tris base (Sigma-Aldrich, Germany) for determination of the optical density (*λ* = 540 nm) in a computer-interfaced, 96-well Synergy H4 (BioTek Instruments USA) photometer microtiter plate reader (Sidoryk et al. 2012).

### MTT cytotoxic test

Twenty microliter of MTT solution (MTT: 3-(4,5-dimethylthiazol-2-yl)-2,5-diphenyltetrazolium bromide, stock solution: 5 mg/ml) was added to each well and incubated for 4 h. After the incubation time was complete, 80 µl of the lysis mixture was added to each well (lysis mixture: 225 ml dimethylformamide, 67.5 g sodium dodecyl sulfate and 275 ml of distilled water). The optical densities of the samples were read after 24 h on a Synergy H4 (BioTek Instruments USA) photometer microtiter plate reader at 570 nm (Sidoryk et al. 2012). All of chemicals were obtained from Sigma-Aldrich, Germany.

### Antiparasitic activity

#### Cell culture

The epithelial cell line VERO (ATCC-Catalog No. CCL-81^TM^), derived from the kidney of the African green monkey, *Cercopithecus aethiops* and fibroblast cell line L-929 (ATCC-Catalog No. CCL-1^TM^), derived from *Mus musculus* (http://www.atcc.org) were grown in Iscoves’s Modified Dulbecco’s Medium (IMDM) supplemented with 10% (v/v) fetal bovine serum (FBS), plus 2 mM l-glutamine, 100 U/ml penicillin, 100.00 µg/ml streptomycin, 5 × 10^−5^ M 2-mercaptoethanol and maintained at 37 °C in a 10% CO_2_ atmosphere. Subcultures were performed via enzymatic cell dissociation in 0.25% trypsin. After dissociation, the cells were placed in culture medium at 4 °C with 10% FBS to inhibit the action of trypsin. The isolated cells were centrifuged for 10 min at 1000×*g* at 4 °C, and the cells were grown in new bottles. This procedure was repeated when cells reached a confluence of ~90%. All commercial reactants and solvents were purchased from either Sigma-Aldrich Laborchemikalien GmbH (Dzitko et al. 2014).

### Preparation of compounds and commercial antibiotics

Suspensions of the compounds **4a**–**l** were freshly prepared (5 mg/ml DMSO) before the cells were exposed, and diluted to appropriate concentrations; 0.9, 1.8, 3.9, 7.8, 15.6, 31.2, 62.5, and 125.00 μg/ml with the culture medium. Cells treated with 2.5% DMSO-solvent served as a control in each experiment.

Stock solutions of the Sulfadiazine (100 mg/ml 1 N NaOH; Catalog No. S8626) was freshly prepared before each experiment, and diluted to appropriate concentrations: 5.00, 25.00, 50.00, 125.00, 250.00, 500.00, 1250.00, and 2500.00 μg/ml, respectively in IMDM complete medium. All commercial reactants and solvents were purchased from either Sigma-Aldrich Laborchemikalien GmbH.

### Cell viability assay

The effects of tested compounds on the viability of mouse fibroblasts L929 and human epithelial VERO cells were evaluated using the MTT assay. The MTT assay was used according to international standards: ISO 10993-5:2009 (Tests for in vitro cytotoxicity; http://www.iso.org/iso/catalogue_detail.htm? csnumber = 36406). L929 and VERO cells were plated into 96-well plates at a density of 1.0 × 10^4^/100 μl/well in culture medium and allowed to attach for 24 h before treatment. Afterwards, culture medium in the plates was replaced by 100 μl compounds suspension at concentration of 0–125 μg/ml and the cells were exposed for 24 h. Then 1 mg/ml MTT (50 μl/well) was added to each well and incubated at 37 °C, 10 % CO_2_ for 2 h. Mitochondrial dehydrogenases of viable cells reduce the yellowish water-soluble MTT to water-insoluble formazan crystals, which were solubilized with dimethyl sulfoxide (DMSO). The cell culture medium was aspirated cautiously, after which 150 μl DMSO was added to each well and mixed thoroughly. Optical density (OD) was read on the ELISA reader (Multiskan EX, Labsystems; http://www.mtxlsi.com/multiskan_EX.htm) at 550 nm. The results were expressed as percentage viability compared with the treated 2.5% DMSO controls. All experiments were performed in triplicate.

### Parasites

Tachyzoites of *T. gondii*, BK strain, were maintained in female C57BL/6 (H-2^b^) and BALB/c (H-2^d^) mice (with genetically determined high and low susceptibility, respectively to *Toxoplasma* infection) via serial intraperitoneal inoculation of 10^5^–10^6^ parasites per mouse in phosphate-buffered saline (PBS). After 48 to 96 h, parasites were harvested via peritoneal lavage using PBS, pH 7.2, and centrifuged at 200×*g* for 5 min to remove blood cells and cell debris. The supernatant containing tachyzoites was collected and centrifuged again at 2000×*g* for 15 min. The final pellet was resuspended in IMDM, quantified in a Burker’s chamber, and used in experimental assays. Inbred mice were kept under standard laboratory conventional conditions. All experimental procedures were conducted according to guidelines of the 9. Local Ethics Commission for Experiments on Animals in Lodz.

### Influence of diaminotriazines on *T. gondii* tachyzoites proliferation

VERO, (2 × 10^4^ cells/100 µl/well) were grown in complete medium (IMDM) on 96-well plates. After 24 h incubation, a medium was removed and then *T. gondii* RH tachyzoites, suspended in culture medium supplemented with 0.9–125.00 μg/ml diaminotriazines **4a**–**l** and as a control-sulfadiazine (5.0–2500.0 μg/ml), were added (2 × 10^5^ tachyzoites/200 µl/well) to the cell monolayers. After subsequent 48 h incubation 1 µCi/well of [^3^H] uracil (Moravek Biochemicals Inc., Brea, CA, USA) was applied to each microculture for further 18–20 h. The amount of the isotope incorporated into the parasite nucleic acid pool, corresponding to the parasite growth, was measured by liquid scintillation counting with 1450 Microbeta Plus Liquid Scintillation Counter (Wallac Oy, Turku, Finland). The cpms of host cells alone (below 250/microculture) were subtracted from cpms of *T. gondii* infected microcultures (Dzitko et al. 2014).

### Spectroscopy

The UV absorption spectra were recorded on T60U spectrophotometer (PG Instruments) equipped with quartz cells of 1 cm path length; the pH value of the solutions were determined with CP-501 pH-meter (Elmetron).

ctDNA, EB and Tris were obtained from the Sigma-Aldrich Company. Tris-HCl buffer solution (concentration 10 mM) was prepared by dissolving solid substance in doubly distilled water and acidify by HCl to pH 7.4. The stock solution of ctDNA was prepared by dissolving solid substance in Tris-HCl buffer. EB solution was prepared by dissolving solid substance in ethanol and Tris-HCl solution. All solutions were stored at 4 °C. The concentrations of ctDNA and EB were determined by absorption spectroscopy using the molar extinction coefficient of 6600 M^−1^ cm^−1^ at 260 nm and 5800 M^−1^ cm^−1^ at 480 nm, respectively. The solutions of ctDNA had a ratio of UV absorbance at 260 and 280 nm larger than 1.8, which indicated that ctDNA was sufficiently free from protein. The stock solutions of substances of **4a**–**l** series at concentration 100 mM were prepared by dissolving solid substance in ethanol and Tris-HCl solution (1:10) (Charak et al. 2012).

### Automated docking setup

Flexible docking was performed by means of the FlexX program (Kramer et al. 1999) as implemented in LeadIT software package (LeadIT 2012). The energies of binding of the triazine derivatives to the active sites of human DNA topoisomerases were analysed using the following crystal structures deposited in the Protein Data Bank: 1SEU (hDNA topoI in complex with indolocarbazole), 3QX3 (hDNA topoII in complex with etoposide; chains A and D), 1ZXM (hDNA topoIIα in complex with AMP-PNP), 4M6K (human DHFR enzyme), 1LII (adenosine kinase), 2O2S (enoyl-acyl carrier reductase), 3AU9 (1-deoxy-d-xylulose-5-phosphate reductoisomerase), 3MB8 (purine nucleoside phosphorylase), and 4M84 (calmodulin-domain protein kinase 1). The native ligands within the active sites, the indolocarbazole and the etoposide, respectively, were removed. In the case of docking simulation within TopoII binding site, two water molecules (HOH-1376-A and HOH-1461-A) were allowed. The active sites were defined to include all atoms within 6.5 Å radius of the native ligands. The first 100 top ranked docking poses were saved for each docking run. For all compounds their protonated forms were considered, as recommended by FlexX program.

### Quantum mechanical calculations

Geometrical parameters of the investigated complexes were optimized within the density functional theory (DFT) approximation employing the B3LYP functional and the 6–311 G** basis set. The corresponding vibrational frequencies were evaluated at the same level of theory. Interaction and binding energies were calculated using the DFT method with the M06-2× exchange-correlation functional and the 6-311 + + G** basis set, employing the supermolecular approach and counterpoise correcting the results. All calculations were carried out using the Gaussian 09 program (Frisch et al. 2009).

## Results and discussion

### Chemistry

**Scheme 1:**
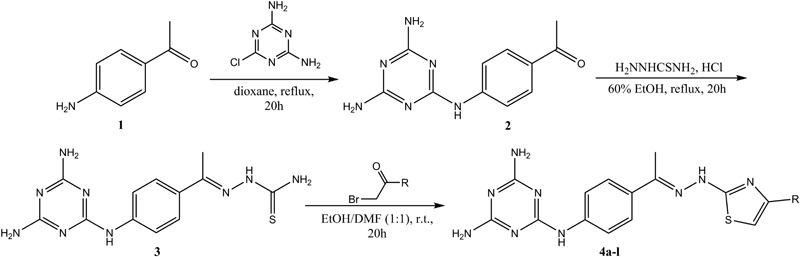
Diaminotriazine-thiazole derivatives **4a**–**l**

The synthetic pathway adopted for the synthesis of the target 2,4-diaminotriazine-thiazole derivatives is presented in Scheme 1. In the first step appropriate 1-(4-(4,6-diamino-1,3,5-triazin-2-yloamino))phenyl)ethanone (**2**) was prepared by alkylation reaction of 1-(4-aminophenyl)-2-chloroethanone (**1**) with 2-chloro-4,6-diamino-1,3,5-triazine in dry dioxane under reflux, with high yield 93% (Scheme 1). Next, (2*E*)-2-(1-(4-((4,6-diamino-1,3,5-triazin-2-yl)amino)phenyl)ethylidene)-hydrazinecarbothioamide (**3**) was synthesized by heating of 1-(4-(4,6-diamino-1,3,5-triazin-2-yloamino))phenyl)ethanone (**2**) and thiosemicarbazide in 60% ethyl ethanol in the presence of concentrated hydrochloric acid with 73% yield. In the next step, a series of target diaminotriazine-thiazoles **4a**–**l** were synthesized by Hantzsch thiazole synthesis between different bromoacetophenones and the hydrazinecarbothioamide **3** in EtOH/DMF (1:1) mixture, with high yield (46–93%) in order to explore the SARs of these derivatives and to obtain potential leading compounds. All of the synthesized derivatives were purified and their structures were characterized by spectroscopic methods ^1^H NMR (700 MHz) and ^13^C NMR (100 MHz), FAB(+)-MS and elemental analyses. ^1^H NMR spectra of thiazoles showed singlet at *δ* (7.20–7.81) due to thiazole-5H proton, which confirms the conversion of substrates into the expected products, and singlet at *δ* (11.10–11.28) indicating the presence of hydrazine NH proton. Also NH_2_ and NH groups attached to the triazine ring give the characteristic peaks, around 6.5 and 9 ppm, respectively. Also ^13^C NMR of carbon atoms present in C=N group resonates around 170 ppm which fully confirms that the condensation between hydrazinecarbothioamide **3** and bromoketones was successful. The [M^+^ + 1] peaks were observed in the mass spectra of all compounds, confirming the assigned structures. Purity of the products was confirmed by the elemental analyses, whose results were in good agreement with the calculated values. All reactions were repeated at least two times and are fully reproducible.

### Antiproliferative activity

The in vitro antiproliferative activity studies of compounds **4a**–**l** against selected cancer cell lines (MV4-11, MCF-7, and A549) and normal mouse fibroblast (Balb/3T3) using *cis*-platin as positive control are listed in Table 1. In our investigation, compounds with IC_50_ below 4 µg/ml are consider as potential drugs (Geran et al. 1972). We started our research study from testing all compounds against biphenotypic B myelomonocytic leukemia MV4-11 cells. Compounds **4a**–**k** showed very high activity against MV4-11 cell line with IC_50_ values between 1.13 and 3.21 µg/ml. In this test only compound **4l** showed no activity. Additionally the cytotoxic activity of compound **4a**–**k** against normal mouse fibroblast Balb/3T3 cells is about 20–100 times lower than against cancer cell lines. Selected eleven highly active compounds were next tested against cancer cell lines MCF-7 and A549. According to our results, compounds **4a**–**4e** and **4g**–**4k** have high to very strong activity against human breast carcinoma MCF-7, with IC_50_ values from 3.18 to 8.72 µg/ml. Compounds **4a**–**4e**, **4h**, and **4i** show moderate activity against human lung carcinoma A549, with IC_50_ 23.76–62.37 µg/ml. Compounds **4f**, **4g**,** 4j**, and **4k** have no antiproliferative activity against A549 cell line.

Next, we have decided to correlate the chemical structure of the triazine-thiazole derivatives with the resulting half maximal inhibitory concentration (IC_50_) value. As can be easily noticed, the highest activity is observed in the case of compounds possessing 4-iodophenyl-, 4-chlorophenyl-, 3,4-dichlorophenyl-, and phenylmethanesulfonamide- substituents. Within the tested series, compound **4c** containing electron-donating methyl group also shows high activity. However, there are no definite differences between substitution effects and activity for each type of cancer. It can be stated, that the antiproliferative activity of the derived compounds is increasing in series MV4-11>MCF-7>A549.

### Anti-*Toxoplasma gondii* activity

In the next step of our studies, the prepared diaminotriazine-thiazoles **4a**–**l** have been used to study intensity of *Toxoplasma gondii* BK strain intracellular proliferation [%] in the VERO host cells. For this purpose, *Toxoplasma gondii* (tachyzoites) of BK strain were incubated with different concentrations of the diaminotriazine-thiazoles **4a**–**l** ranging from 0.9 to 125 μg/ml. The parasite growth inhibition was monitored by measuring the specific incorporation of [^3^H]uracil in the parasite’s nucleic acids. The percentages of the parasite proliferation in VERO host cells by the compounds **4a**–**l** and the control drug-sulfadiazine, as well as IC_50_ values are summarized in Tables 2 and 3. According to these results, all diaminotriazines **4a**–**l** showed significant anti-*Toxoplasma gondii* activity, with IC_50_ values 9–68 times lower than those observed for sulfadiazine (IC_50_ = 1024.69 μg/ml). The structure–activity relationship (SAR) study revealed that diaminotriazine-thiazole derivatives **4f**, **4g**, **4i**, and **4l** with the 3,4-dichlorophenyl-, 4-acetylaminophenyl-, 2-oxochromenyl- and benzo[d]oxazolyl- substituents were found to be the most potent anti-*Toxoplasma gondii* agents, with an IC_50_ of 14.95–23.09 μg/ml. Adding a second chlorine atom to position 3 increases the activity of the resulting compound **4i** three times. It can be easily seen that replacing the fluorine atom in compound **4a** with bioisosteric methyl group does not change the activity of the resulting compound **4c**. The lowest activity showed compound containing 4-nitrophenyl group (IC_50_ = 118.98 μg/ml), however, it was still at least nine times lower than the value obtained for sulfadiazine.

**Table 2:** Effect of studied compounds on the intensity of *Toxoplasma gondii* BK strain intracellular proliferation [%] in the VERO host cells

Triazines	Concentration (µg/ml)								IC_50_
	0.9	1.8	3.9	7.8	15.6	31.2	62.5	125.0	(µg/ml)
**4a**	102.46	101.36	101.36	107.78	99.12	77.64	34.92	17.60	51.63
**4b**	100.43	105.92	100.69	98.25	74.61	52.20	26.15	14.70	33.03
**4c**	101.56	102.36	109.87	99.95	86.17	58.62	40.11	30.93	51.38
**4d**	101.71	103.59	101.58	105.62	75.19	62.78	29.30	15.14	37.81
**4e**	102.78	103.58	99.87	92.80	80.70	75.10	48.67	14.61	49.18
**4f**	101.02	98.89	75.89	64.30	55.65	50.52	37.17	14.03	21.53
**4g**	100.01	100.69	69.35	66.62	46.71	42.95	41.74	19.11	18.70
**4h**	102.74	105.09	113.25	100.59	85.65	77.46	74.04	39.38	118.98
**4i**	100.65	102.36	78.96	55.89	45.69	41.14	nt	nt	14.95
**4j**	99.93	100.25	99.58	89.65	78.25	67.01	46.07	34.60	59.87
**4k**	100.41	100.46	96.35	85.92	63.58	49.35	36.07	17.72	31.51
**4l**	99.98	101.25	106.91	79.89	56.46	45.15	20.34	nt	23.09
**3**	99.98	106.58	98.58	84.21	64.59	57.78	49.44	48.71	70.37

To calculate the intensity of *T. gondii* proliferation compared to the untreated blank, the Equation was used: Proliferation (%) = [100 × *sample cpm /**blank cpm], *sample cpm—the mean value of the measured [^3^H] uracil incorporation into DNA of *Toxoplasma* tachyzoites, corresponding to the parasite growth in VERO cells treated with **4a**–**l** compounds from 0.9 to 125.0 µg/ml, **blank cpm—the mean value of the measured [^3^H] uracil incorporation into the tachyzoites of the untreated VERO cells; IC_50_: represents the concentration of tested compounds that was required for 50% inhibition of *T. gondii* proliferation in vitro. IC_50_ values were determined based on the plotted curves using GraphPad Prism program (version 6.04). The results of the experiments are shown as mean arithmetic values from nine repeats (three independent experiments)

*nt* not tested

**Table 3:** Effect of sulfadiazine on the intensity of *Toxoplasma gondii* BK strain intracellular proliferation (%) in the VERO host cells

Sulfadiazine	
Concentration	Proliferation
(µg/ml)	(%)
0.00	100.27
5.00	91.97
25.00	80.47
50.00	70.67
125.00	55.16
250.00	52.79
500.00	41.60
1250.00	39.71
2500.00	39.64
IC_50_ 1024.69 µg/ml	

To calculate the intensity of *T. gondii* proliferation compared to the untreated blank, the Equation was used: Proliferation (%) = [100 × *sample cpm /**blank cpm], *sample cpm—the mean value of the measured [^3^H] uracil incorporation into DNA of *Toxoplasma* tachyzoites, corresponding to the parasite growth in VERO cells treated with Sulfadiazine, **blank cpm—the mean value of the measured [^3^H] uracil incorporation into the tachyzoites of the untreated VERO cells; IC_50_: represents the concentration of tested compounds that was required for 50% inhibition of *T. gondii* proliferation in vitro. IC_50_ values were determined based on the plotted curves using GraphPad Prism program (version 6.04). The results of the experiments are shown as mean arithmetic values from nine repeats (three independent experiments)

### Cytotoxicity against mouse L929 fibroblast and human VERO cells

In our research, we aimed at obtaining compounds inhibiting the parasite growth at possibly low concentrations and simultaneously exhibiting low toxicity on the host cells. Thus the next stage of our investigation was determination of the toxicity of the newly synthesized compounds. If the compounds exhibit toxicity effects it is the basis for its disqualification from clinical trials. In order to prove that the newly obtained compounds are useful for further clinical studies, we decided to investigate the cytotoxic effects of diaminotriazine-thiazoles **4a**–**l** on mouse L929 fibroblast, as well as the humane VERO cells using an MTT assay. The results of the cytotoxicity (CC_30_) and anti-*Toxoplasma gondii* activity studies, presented in Tables 4 and 2 respectively, show that in all cases the parasite growth was inhibited at concentrations non-cytotoxic for the host cells. These studies also confirm the earlier results for Balb/3T3 during antiproliferative activity tests (Table 1). We can conclude that the cytotoxic effects of compounds **4a**–**l** against mouse L929 fibroblast, as well as the humane VERO cells is about 1–8 times lower than against *Toxoplasma gondii* parasite.

**Table 4:** Cytotoxic effect of studied compounds on mouse L929 fibroblast (1) and human VERO cells (2)

Triazines	L929/VERO	Concentration (µg/ml)								CC_30_
		0.9	1.8	3.9	7.8	15.6	31.2	62.5	125.0	(µg/ml)
**4a**	1	96.56	101.11	104.82	98.99	102.39	110.77	115.32	110.86	>125.0
	2	90.45	97.57	100.07	95.93	103.14	107.23	105.21	99.97	
**4b**	1	94.75	95.28	99.43	99.43	110.47	112.67	118.54	117.97	>125.0
	2	93.53	95.79	90.06	94.73	103.48	114.83	115.17	113.05	
**4c**	1	98.20	95.55	98.15	92.90	101.77	105.21	106.14	118.32	>125.0
	2	92.37	101.89	98.96	101.17	99.59	101.51	112.38	108.67	
**4d**	1	99.92	97.75	99.92	93.69	108.48	112.76	115.23	113.20	>125.0
	2	99.87	94.44	90.06	86.03	104.01	113.67	100.45	112.00	
**4e**	1	95.95	97.62	99.08	88.84	98.81	107.95	108.96	100.54	>125.0
	2	100.31	100.31	97.04	87.32	101.03	113.39	117.04	112.86	
**4f**	1	98.99	102.21	101.95	96.25	100.89	104.60	109.54	108.66	>125.0
	2	97.81	100.84	104.30	104.39	108.19	117.52	103.77	111.27	
**4g**	1	102.34	102.12	100.36	98.86	94.31	99.83	103.01	105.39	>125.0
	2	93.72	90.59	95.35	98.14	98.43	98.96	94.25	91.41	
**4h**	1	100.23	98.46	100.36	104.86	105.30	113.91	117.53	103.62	>125.0
	2	104.39	99.44	106.41	109.35	110.02	109.30	109.11	103.91	
**4i**	1	100.67	97.89	96.08	100.23	98.11	108.17	100.50	nt	>62.5
	2	97.52	93.67	93.86	92.61	97.33	111.51	111.01	nt	
**4j**	1	98.90	106.36	104.20	101.86	100.71	104.07	102.92	106.67	>125.0
	2	79.82	93.81	93.62	89.25	90.98	96.22	97.76	89.44	
**4k**	1	108.85	106.76	106.01	100.60	105.67	110.79	115.51	129.97	>125.0
	2	91.94	95.55	99.34	95.74	104.20	105.74	112.81	113.15	
**4l**	1	103.88	109.00	103.03	101.74	105.02	105.52	108.10	nt	>62.5
	2	97.13	87.85	95.16	91.03	96.08	95.40	97.33	nt	
**3**	1	110.29	115.71	110.83	105.67	105.47	111.83	111.48	110.19	>125.0
	2	97.23	93.96	97.28	88.77	89.49	96.22	95.07	89.15	

To calculate the reduction of host cells (L929 and VERO) viability compared to the untreated blank, the Equation was used: Viability (%) = [100 × *sample OD_570_ /**blank OD_570_], *sample OD_570_—the mean value of the measured optical density, corresponding to the MTT reduction by metabolically active cells to form an insoluble purple formazan product that is quantifiable by spectrophotometry, after treatment with 1–13 compounds from 0.9 to 125.0 µg/ml, **blank OD_570_—the mean value of the measured optical density of the untreated cells; IC_30_: represents the concentration of tested compounds that was required for 30% proliferation inhibition in vitro. The effect of tested compounds on the cell lines was measured using MTT assay according to the international standard: ISO 10993-5:2009(E). The results of the experiments are shown as mean arithmetic values from nine repeats (three independent experiments)

*nt* not tested

### Molecular modeling studies

In order to find and explain the possible mechanism of anticancer activity of 1,3,5-triazine-based compounds (**4a**–**l**) the molecular docking calculations were performed. Dihydrofolate reductase (DHFR), the first enzyme used in our in silico experiments, plays an important role in nucleic acid synthesis and is targeted by some anticancer agents (Schweitzer et al. 1990). The mentioned enzyme catalyzes the NADPH-dependent reduction of dihydrofolate to tetrahydrofolate (THF). It was also showed that some of 1,3,5-triazine derivatives acted as DHFR inhibitors, and thus, they inhibited the growth of cancer cells. For example, triazine-benzimidazole hybrids were tested on more than 60 cancer cell lines showing broad spectrum of anticancer activity (Singla et al. 2015). Moreover, such compounds turned out to be strong DHFR inhibitors with IC_50_ values in the low micromolar range. Results from docking simulations demonstrated that 1,3,5-triazine ring of the triazine-benzimidazole hybrids strongly interacts (*π*–*π* stacking) with Trp-624 located in the active site of human DHFR. In turn, Balaha and co-workers (Balaha et al. 2016). synthesized different 2,4,6-trisubstituted 1,3,5-triazine derivatives that inhibited the growth of A549 cells (lung cancer) stronger than methotrexate. Also in that case the presence of triazine ring was crucial for inhibition of DHFR. Bearing all this in mind, we have conducted docking of our novel compounds **4a**–**l** into human DHFR enzyme (PDB id: 4M6K) using a FlexX docking software implemented in LeadIT package. Contrary to what we expected, it was found that all of the investigated derivatives displayed relatively weak affinities to the active site of human DHFR. Docking score values calculated for compounds **4a**–**l** ranged from −25.23 to −31.85 kcal/mol, while the docking score for native ligand (i.e., folic acid) was −64.76 kcal/mol (Table 5). The observed difference in affinity of the synthesized compounds and folic acid towards human DHFR could result from the differences in the number of hydrogen bonds formed inside the binding. Thus, one should rather exclude that anticancer activity of **4a**–**l** results from inhibition of human DHFR.

**Table 5:** Docking scores for compounds **4a**-**l** docked into the active sites of anticancer and anti-toxoplasmosis molecular targets

Targets	Docking scores (kcal/mol)												Native ligand
	**4a**	**4b**	**4c**	**4d**	**4e**	**4f**	**4g**	**4h**	**4i**	**4j**	**4k**	**4l**	
4M6K	−31.85	−28.07	−28.43	−28.22	−28.39	−28.03	−29.42	−30.61	−25.23	−28.36	−30.19	−26.92	−64.76
1SEU	−41.66	−41.64	−41.90	−41.47	−41.26	−45.31	−41.14	−41.45	−37.23	−41.04	−36.90	−41.60	−36.65
3QX3(A)	−32.52	−29.87	−29.86	−29.87	−29.86	−39.32	−30.93	−33.55	−34.94	−34.38	−38.54	−38.85	−16.62
3QX3(D)	−38.98	−37.24	−36.64	−36.90	−36.50	−41.03	−38.34	−37.61	−36.88	−37.44	−37.93	−41.32	−37.29
1ZXM	−30.05	−26.18	−35.22	−27.14	−40.48	−27.66	−30.62	−29.43	−28.25	−26.18	−25.54	−26.16	−78.76
1LII	−18.71	−13.54	−15.32	−13.58	−12.52	−19.15	−16.02	−18.00	−13.84	−12.06	−24.93	−19.96	−58.84
2O2S	−21.66	−10.56	−11.20	−20.38	−6.52	−18.72	−11.45	−16.46	−13.59	−10.24	−23.60	−18.35	−19.55
3AU9	−15.38	−17.53	−17.54	−17.54	−17.53	−23.07	−21.85	−18.89	−17.38	−16.83	−17.01	−17.46	−44.74
3MB8	−31.24	−26.02	−31.46	−26.00	−23.14	−30.52	−28.79	−28.77	−33.69	−26.98	−33.73	−33.13	−42.89
4M84	−20.17	−15.64	−19.79	−15.62	−15.62	−23.61	−17.42	−16.68	−17.54	−21.32	−29.16	−24.38	−26.55

**Fig. 1:**
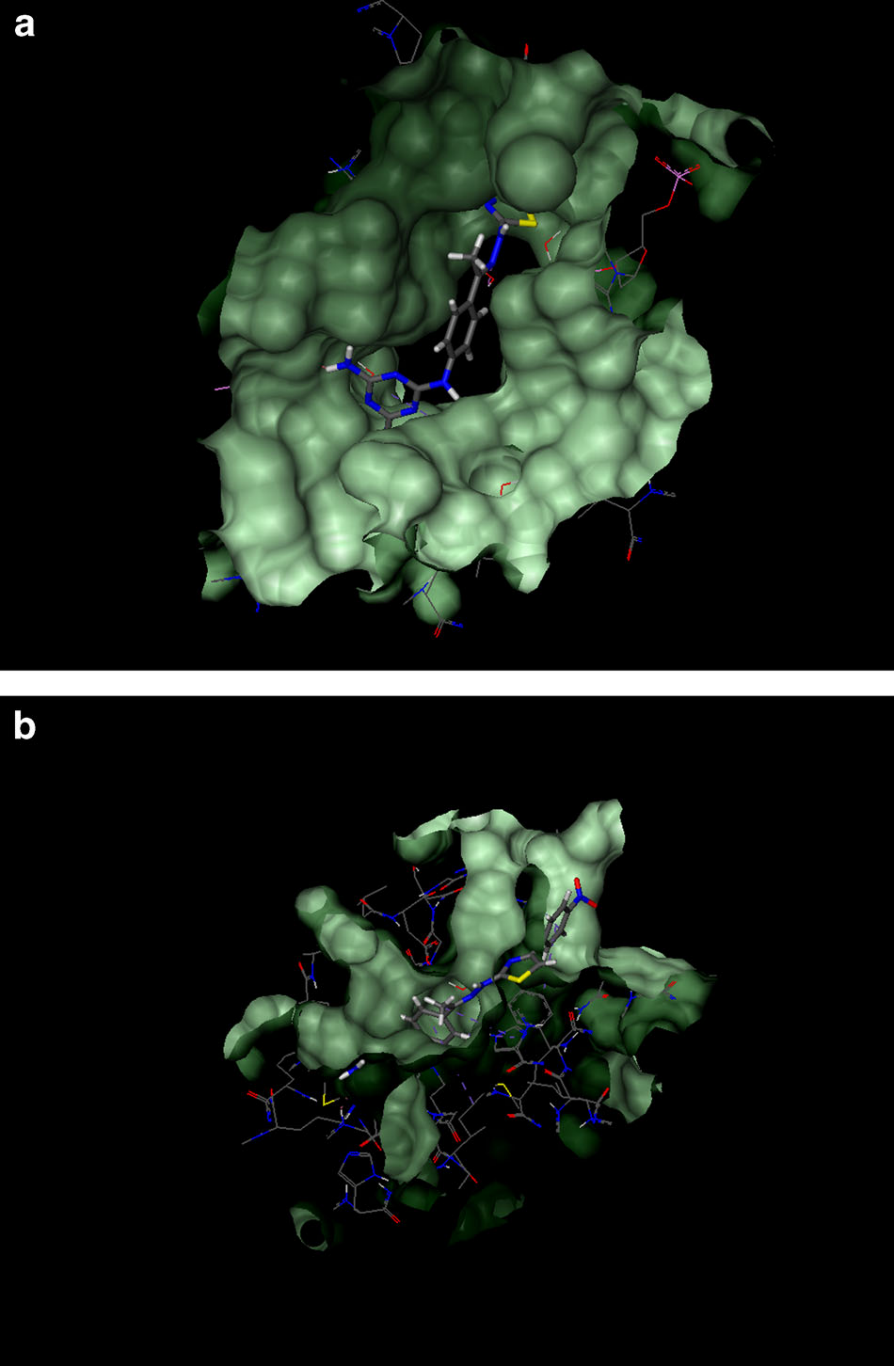
Binding pocket located on the chain A of hDNA topoIIα (3QX3) with **4f** (**a**); **4h** in the binding pocket of purine nucleoside phosphorylase (3MB8) (**b**)

In the next step, we investigated whether compounds **4a**–**l** might block the action of human DNA topoisomerases (hDNA topo), i.e., the enzymes that are involved in regulation of the metabolism and topology of DNA (Pommier et al. 2016). The energies of binding of the triazine derivatives to the active sites of human DNA topoisomerases were analysed using the following crystal structures deposited in the Protein Data Bank: 1SEU (hDNA topoI in complex with indolocarbazole), 3QX3 (hDNA topoII in complex with etoposide; chains A and D), 1ZXM (hDNA topoIIα in complex with AMP-PNP). The literature search reveals that 1,3,5-triazine derivatives may affect the activity of both human topoI and topoII enzymes. Nakamura et al. (Nakamura et al. 2011). showed that, depend on the substitution pattern, some of the carborane conjugated triazines completely inhibited human DNA topoisomerase I (without affecting topoII activity) or acted as selective topoII inhibitors. In turn, ruthenium complexes of triazine-based compounds obtained by Du and co-workers (Du et al. 2014) exhibited dual inhibition of human topoI and topoII. There were also synthesized such derivatives, including 4-amino-6-phenylamino-1,3,5-triazines and 4,6-disubstituted 1,3,5-triazin-2(1*H*)-ones, which catalytically and selectively inhibited htopoIIα through binding to its ATP-dependent subunit (Pogorelčnik et al. 2014, 2015). As can be seen from (Table 5) compounds **4a**–**l** were characterized by particularly beneficial docking scores for the DNA-dependent subunits of both topoI and topoII enzyme models (1SEU, 3QX3). Specifically strong interactions were observed between the investigated triazines and the binding pocket located on the chain A of hDNA topoIIα (3QX3). Affinities of compounds **4a**–**l** towards 3QX3 (A), expressed as their respective docking scores, were much higher than that of etoposide (e.g., −39.32 kcal/mol for **4f** vs. −16.62 kcal/mol for etoposide) (Fig. 1). On the other hand, taking into account the docking scores calculated for the title compounds docked to ATP-binding site of hDNA topoIIα (1ZXM), it can be concluded the possibility of inhibition of the topoisomerase IIα through inhibition of its ATP-ase activity is rather low. Unfortunately, there was no correlation between the docking scores calculated for **4a**–**l (**even for 1SEU and 3QX3 enzyme models) and their anticancer activity. This should not be particularly surprising for at least two reasons: (i) docking scores are correlated rather with affinity towards specific molecular targets, (ii) overall anticancer activity of compound depends not only from its interaction with molecular target(s). Summarizing, out of the investigated enzymes the strongest binding is expected for 1SEU and 3QX3. Thus, these enzymes seem to be the most probable molecular targets for the triazine-based compounds **4a**−**l**.

Our in-depth studies on the pharmacological activity of 2,4,6-trisubstituted 1,3,5-triazine derivatives (**4a**−**l**) revealed that these compounds possessed also anti-toxoplasmosis effect. To better understand the molecular basis of this type of activity, we analysed the binding affinities of the compounds to the active sites of the following enzymes of *Toxoplasma*: 1LII (adenosine kinase), 2O2S (enoyl-acyl carrier reductase), 3AU9 (1-deoxy-d-xylulose-5-phosphate reductoisomerase), 3MB8 (purine nucleoside phosphorylase), and 4M84 (calmodulin-domain protein kinase 1). Following the results from (Table 5), it was observed that none of the compounds tested were characterized by docking scores clearly correlated with anti-toxoplasmosis activity. Such phenomenon do not exclude the possibility that triazine derivatives **4a**−**l** can effectively interact with the enzymes used in the docking experiments. For instance, in the case of enoyl-acyl carrier reductase (2O2S), the highest predicted affinity was observed for derivatives **4a**, **4d**, and **4k** (−21.66, −20.38, and −23.60 kcal/mol, respectively). At the same time the docking score for native inhibitor of 2O2S (i.e., triclosan) was −19.55 kcal/mol. Interestingly, the most active compound **4i** (IC_50_ = 14.95 µg/ml) was characterized by relatively low affinity. Taking into account the differences between the docking scores for compounds **4h** (displaying the weakest bioactivity) and **4i** (having the strongest bioactivity), as well as comparing their docking scores with the strength of binding of native ligands, the purine nucleoside phosphorylase (3MB8) was selected as the probable molecular target.

### Spectroscopic properties

DNA performs significant functions in living cells because it encodes information on protein and enzymes synthesis through the process of replication and transcription of genetic information. Therefore, DNA is quite often the main cellular target for the interaction studies of anticancer agents. Generally, the interactions of small molecules with DNA involve three binding modes: intercalation, groove binding, and interaction on the outside of the helix (Zhang et al. 2011; Rafique et al. 2013). In order to thoroughly understand the mechanism of interaction of the newly derived compounds with ctDNA, the spectroscopic studies were divided into four phases. We measured, (i) UV–Vis spectra of newly obtained compounds **4a**–**l**, (ii) absorbance spectra of the solutions containing a constant concentration of ctDNA and increasing amounts of **4a**–**l** derivatives, (iii) absorbance spectra of the solutions containing a constant concentration of **4a**–**l** derivatives and increasing ctDNA concentration, and finally, (iv) absorbance spectra of the solutions containing a constant concentration of ethidium bromide (EB), a constant concentration of ctDNA and increasing amounts of the **4a**–**l** derivatives. All spectra were measured immediately after solutions preparation and after an 8 min incubation.

The UV–Vis absorbance spectra of 2,4-diaminotriazine-thiazoles **4a**–**l** (with increasing concentration) in mixture of ethyl alcohol and Tris-HCl (1:10) are shown in Table 6. These compounds, except **4g** and **4l**, in the ultraviolet/visible region exhibit two major absorption bands in the 281–304 (nm) and 320–364 (nm) ranges.

**Table 6:** UV–Vis spectra of the free 2,4-diaminotriazine-thiazoles **4a**–**l**

Triazines	*λ*_1_ (nm)	*λ*_2_ (nm)
**4a**	284	335
**4b**	289	338
**4c**	289	335
**4d**	290	340
**4e**	294	364
**4f**	291	352
**4g**	303	–
**4h**	281	320
**4i**	292	352
**4j**	294	341
**4k**	290	332
**4l**	304	–

**Fig. 2:**
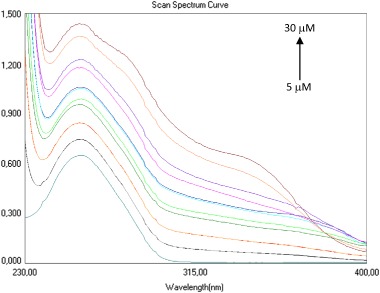
The absorption spectrum of the solution containing 100 mM of DNA and increasing amounts of **4d**

**Fig. 3:**
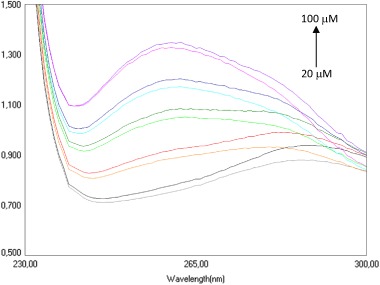
The absorption spectrum of the solutions containing 30 μM of **4e** and increasing amounts of DNA

The example of absorption spectrum of the ctDNA with increasing concentration of the 2,4-diaminotriazine **4d** is shown in Fig. 2. For the whole series of compounds with constant DNA concentration, the hyperchromic effect was observed. For each DNA-diaminotriazine complex, absorption of the mixture at 258 nm showed a decrease in absorbance compared to the sum of the individual components, which clearly shows that the test compounds interact with DNA. It has been found that in general the incubation time does not play a key role in the formation of DNA linkage suggesting a fast superficial compounds bonding to DNA. Compound **4d**, is here an exception, with a noticeable decrease in absorbance (hypochromic shift) for 20 μM after 8 min incubation. The next step of the analysis was investigation of the effect of DNA concentration and time of incubation on the formation of DNA linkage with the test compounds. After adding a constant concentration of individual compounds to the DNA solution, hypsochromic shifts were observed relative to the sum of the absorbances of the individual components (Fig. 3). In the case of compound **4e**, it was noticed that lower DNA concentrations lead to a larger decrease in absorbance of the solutions, which may mean that **4e** binds better at lower DNA concentration. Also it has been found that in the case of compounds **4d** and **4e**, 8 min incubation plays a key role in the formation of DNA linkage.

**Fig. 4:**
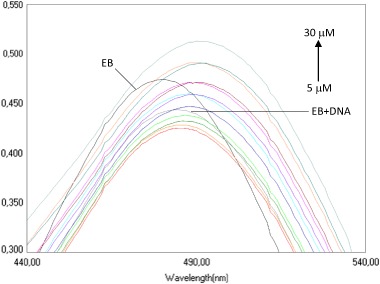
The absorption spectrum of the solutions containing 80 μM EB, 80 μM DNA and increasing concentration of **4f**

The interaction of EB with DNA was studied by many researchers (Srivastava et al. 2013). These studies have proven that the strong mode of binding of EB to DNA results in the intercalation of the planar phenanthridinium ring between adjacent base pairs on the double helix of DNA. To confirm the mechanism of intercalation of newly synthetized compounds, we decided to explore the possibility of displacing EB from its EB-DNA complex. Absorption spectra of all derivatives were recorded using the standard constant concentration of EB and DNA, as well as increasing concentrations of the analysed substances. Observed bathochromic shift (red shift), with decrease in the absorbance (hypochromic shift) indicates intercalation of the 2,4-diaminotriazine-thiazoles **4a**–**l** between the DNA base pairs (Fig. 4) (Pitié et al. 2005). In addition, we observed a hyperchromic shift probably due to the formation of additional hydrogen bonds between two NH_2_ groups and the three nitrogen atoms and DNA bases (Srivastava et al. 2013).

### Calculations

Multiple-point hydrogen bonding is responsible for the formation of base pairs in the double helix of DNA and determines the stability of DNA double helix. Diaminotriazine can form a highly stable complex with two molecules of thymine or uracil interacting through six hydrogen bonds (Yu et al. 2008; Mao and Bong 2015; Zeng et al. 2012). Because of the serious side effects of existing drugs, specific recognition of DNA by drug molecules is a key factor in the design of DNA-target drugs. In the present study, the structure as well as the interaction and binding energies of a model complex formed by compound **4a** and two thymine molecules (denoted as **4a**-**T**_**2**_ in the following) are investigated employing quantum mechanical methods.

**Fig. 5:**
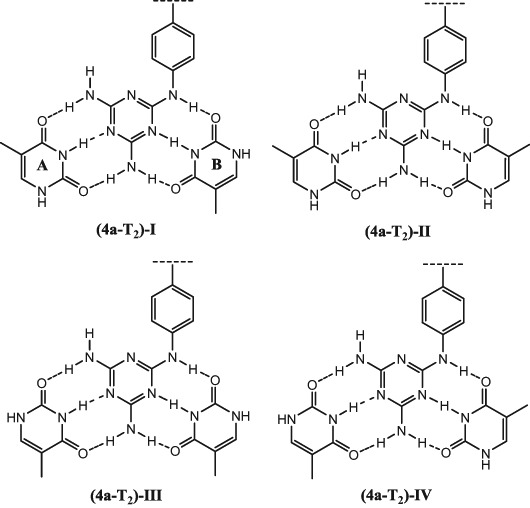
The four investigated orientations of thymine molecules with respect to the diaminotriazine **4a**

The first step of the study was optimization of geometrical parameters of the investigated complex. Four different starting points were used for that purpose, with different mutual orientations of the three subsystems (Fig. 5). In all four cases, to ensure possibly strong interaction between the subsystems, the molecules were oriented in a way allowing formation of six hydrogen bonds. Optimization was carried out employing the DFT method with the traditionally chosen B3LYP exchange-correlation functional and the 6-311G** basis set. To confirm that the resulting structures were stationary points, optimization was followed by calculation of vibrational frequencies within the same approximation. Optimized geometrical parameters are reported in the Supplementary Material.

Next, interaction energy of the complex, Δ*E*(*ABC*), was calculated using the supermolecular approach as the difference between the energy of the complex and the energies of its three components, and the results were counterpoise corrected:

$$\Delta E\left( {ABC} \right) = E_{ABC}^{ABC}\left( {ABC} \right) - \mathop {\sum}\limits_{i\,=\,A}^C {E_{ABC}^{ABC}\left( i \right)}.$$

In the above, *E*_*G*_^*B*^(S) denotes energy of system *S* evaluated at the geometry *G* using basis set *B*. For example, *E*_*ABC*_^*ABC*^(A) is the energy of system *A* calculated at the optimized geometry of the complex *ABC* in the basis set of the complex *ABC*. Additionally, the binding energy of the complex, *E*_bind_(*ABC*), was evaluated:

$$E_{{\mathrm{bind}}}({{ABC}}) = \Delta E\left( {{{ABC}}} \right) + \mathop {\sum}\limits_{i\,=\,A}^C {\left( {E_{{{ABC}}}^i\left( i \right) - E_i^i\left( i \right)} \right)}.$$

Based on the detailed analysis of performance of different exchange-correlation functionals and basis sets carried out in our earlier work (Łączkowski et al. 2014) the M06-2× exchange-correlation functional combined with the 6-311++G** basis set are used in the interaction and binding energies evaluation. All calculations were carried out using the Gaussian 09 package (Frisch et al. 2009).

Among the investigated mutual orientations of the three subsystems, the one denoted as (**4a**-**T**_**2**_)-**II** corresponds to the lowest total energy. The energy of the remaining three orientations increases from (**4a**-**T**_**2**_)-**IV**, through (**4a**-**T**_**2**_)-**I** to (**4a**-**T**_**2**_)-**III**. This ordering corresponds to the Boltzmann populations of the respective complexes equal to approximately 29, 27, 23, and 21% (Table 7). Calculated values of the interaction energy for the four investigated complexes are in the order of −38 kcal/mol, with the differences between them being negligible. Binding energies are in the order of −34.5 kcal/mol, that is approximately 3.5 kcal/mol higher than the corresponding interaction energies. This results from deformation of the geometries when going from the isolated monomers to their complex. Again, differences between the binding energies for the four orientations are very small. Both, interaction and binding energies follow the same ordering as do Boltzmann populations, with the complex constituting the largest part of the total population corresponding to the strongest interaction. Analysis of the selected geometrical parameters presented in (Table 7) shows that hydrogen bond intermolecular distances (denoted as *r*) and angles (denoted as *α*) have very similar values for the four investigated complex orientations, in nice agreement with the calculated values of interaction energies.

**Table 7:** The DFT/B3LYP/6-311++G** selected geometrical parameters, conformer populations *X*, and predicted M06-2×/ 6-311++G** interaction (*ΔE*) and binding (*E*_bind_) energies of the investigated complex. Symbols *N*_A_ and *N*_B_ denote N3-atom in thymine **A** and **B**, respectively. Interatomic distances *r* in Å, angles *α* in deg, populations *X* in %, interaction and binding energies in kcal/mol. (see Fig. 1 in the Supporting Information for the details of hydrogen bond numbering)

Complex	*r*_1_	*α*_1_	*r*_2_	*α*_2_	*r*_3_	*α*_3_	*r*_4_	*α*_4_	*r*_5_	*α*_5_	*r*_6_	*α*_6_	*X*	*ΔE*	*E*_bind_
**(4a**-**T**_**2**_**)**-**I**	2.938	177.0	2.910	179.6	2.994	178.6	2.982	171.7	2.948	178.3	2.964	179.4	23.13	−38.16	−34.53
**(4a**-**T**_**2**_**)**-**II**	2.938	177.0	2.910	179.6	2.993	178.7	2.970	171.6	2.947	179.2	2.974	179.6	28.72	−38.29	−34.64
**(4a**-**T**_**2**_**)**-**III**	2.950	176.6	2.910	179.5	2.982	178.9	2.982	171.6	2.948	178.3	2.965	179.4	21.14	−38.11	−34.49
**(4a**-**T**_**2**_**)**-**IV**	2.951	176.6	2.910	179.5	2.980	178.9	2.969	171.6	2.947	179.2	2.976	179.6	27.01	−38.26	−34.61

## Conclusion

We have developed an efficient method for the synthesis of diaminotriazine-thiazole derivatives. Most of the reported compounds showed very high activity against biphenotypic B myelomonocytic leukemia MV4-11, and human breast carcinoma MCF-7 cell lines with IC_50_ values between 1.13 and 4.28 µg/ml. Additionally the cytotoxic activity of diaminotriazine-thiazoles against normal mouse fibroblast Balb/3T3 cells is about 20–100 times lower than against cancer cell lines. According to our results, diaminotriazine-thiazoles showed significant anti-*Toxoplasma gondii* activity, with IC_50_ values 9–68 times lower than those observed for sulfadiazine. We also showed that the cytotoxic effects of compounds against mouse L929 fibroblast, as well as the humane VERO cells are even eight times lower than against *Toxoplasma gondii* parasite. Molecular docking studies indicated DNA-binding site of hTopoI and hTopoII as possible anticancer targets and purine nucleoside phosphorylase as possible anti-toxoplasmosis target. Our UV–Vis spectroscopic results indicate also that diaminotriazines tend to interact with DNA by intercalation. Calculated values of interaction energy for the four investigated complexes of compound **4a** with two thymine molecules are in the order of −38 kcal/mol, and binding energies are approximately 3.5 kcal/mol higher than the corresponding interaction energies. These highly active compounds characterized by very low toxicity can serve as new lead compounds for the future development of anticancer and anti-toxoplasmosis drugs.

## Electronic supplementary material

Supplementary Information
