# Supplementary material for: Synthesis, molecular docking, ctDNA interaction, DFT calculation and evaluation of antiproliferative and anti-Toxoplasma gondii activities of 2,4-diaminotriazine-thiazole derivatives
Source: Med Chem Res. 2018 Feb 6;27(4):1131–48. doi: 10.1007/s00044-018-2136-6 (PMC5854716; doi:10.1007/s00044-018-2136-6)
Supplement: Supplementary file 1 — Supplementary Information [file 44_2018_2136_MOESM1_ESM.docx]

**Supporting Information**

**Synthesis, molecular docking, ctDNA interaction, DFT calculation and evaluation of antiproliferative and anti-*Toxoplasma gondii* activities of
2,4-diaminotriazine-thiazole derivatives**

**Krzysztof Z. Łączkowski^1,^*, Joanna Anusiak^1^, Marta Świtalska^2^, Katarzyna Dzitko^3^, Joanna Cytarska^1^,**

**Angelika Baranowska-Łączkowska^4^, Tomasz Plech^5^, Agata Paneth^6^, Joanna Wietrzyk^2^, Joanna Białczyk^1^**

*^1^Department of Chemical Technology and Pharmaceuticals, Faculty of Pharmacy, Collegium Medicum, Nicolaus Copernicus University, Jurasza 2, 85-089 Bydgoszcz, Poland,*

*^2^Institute of Immunology and Experimental Therapy, Polish Academy of Sciences, Rudolfa Weigla 12, 53-114 Wrocław, Poland*

*^3^Department of Immunoparasitology, University of Lodz, Banacha 12/16, 90-237 Lodz, Poland*

*^4^Institute of Physics, Kazimierz Wielki University, Plac Weyssenhoffa 11, 85-072 Bydgoszcz, Poland*

*^5^Department of Pharmacology, Faculty of Health Sciences, Medical University of Lublin, Chodźki 4a, 20-093 Lublin, Poland*

*^6^Department of Organic Chemistry, Faculty of Pharmacy, Medical University of Lublin, Chodźki 4a, 20-093 Lublin, Poland*

**Contents**

**S1**. The numbering of the X-Y interatomic distances r_i_ in the X-H-Y hydrogen bonds in the investigated complex (orientation I). The same numbering is used for the angles α_i_ of the X-H-Y bonds, and for all studied mutual orientations of the three subsystems.

**S2**. Optimized structures of complexes **(4a-T_2_)-I**, **(4a-T_2_)-II**, **(4a-T_2_)-III** and **(4a-T_2_)-IV** calculated within the DFT/B3LYP/6-311++G** approximation.

**S3**. Cartesian coordinates of complexes **(4a-T_2_)-I, (4a-T_2_)-II, (4a-T_2_)-III** and **(4a-T_2_)-IV** calculated within the DFT/B3LYP/6-311++G** approximation.

**S1.**


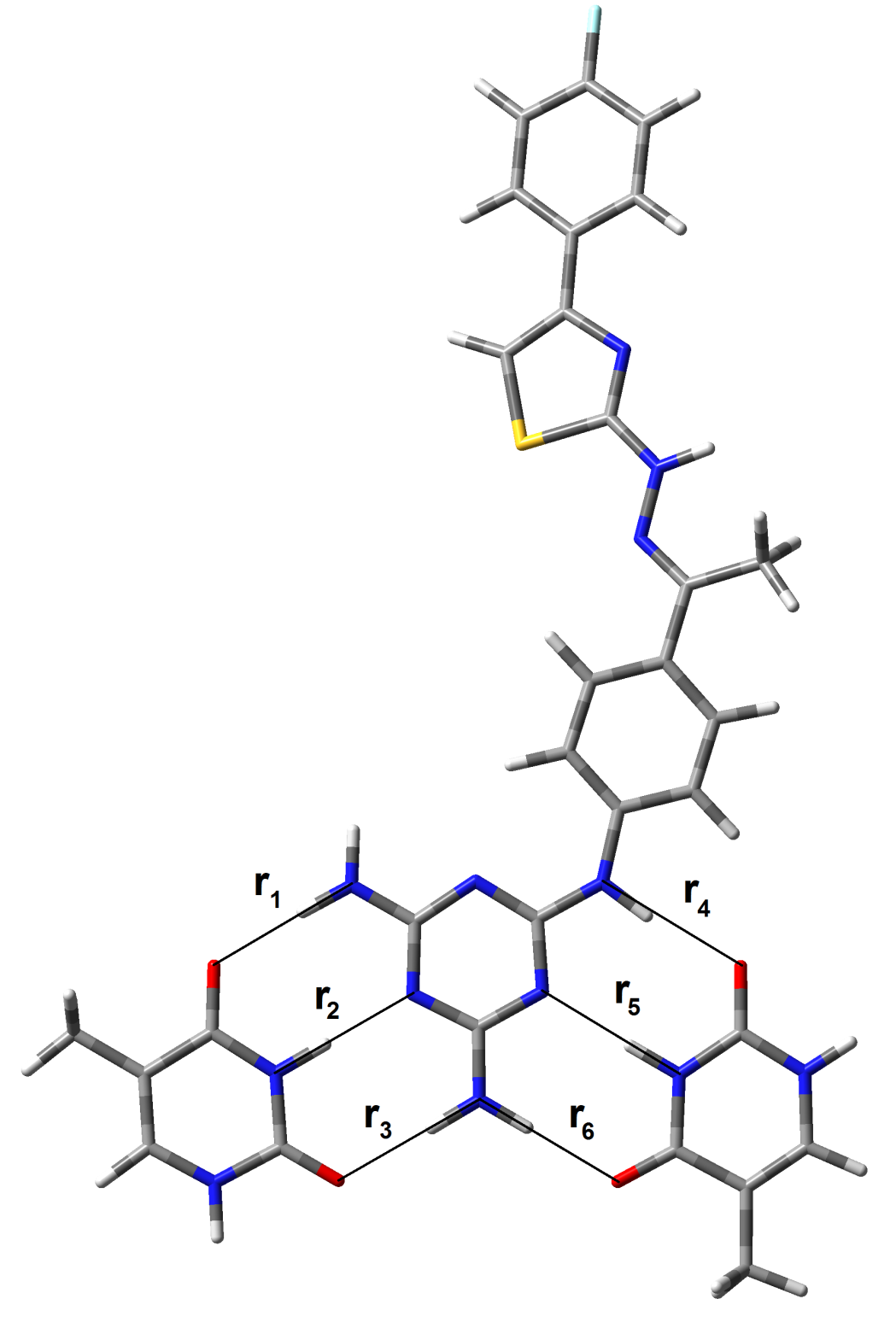


**Figure 1**. The numbering of the X-Y interatomic distances r_i_ in the X-H-Y hydrogen bonds in the investigated complex (orientation I). The same numbering is used for the angles α_i_ of the X-H-Y bonds, and for all studied mutual orientations of the three subsystems.

**S2.**

**(4a-T_2_)-I**

**
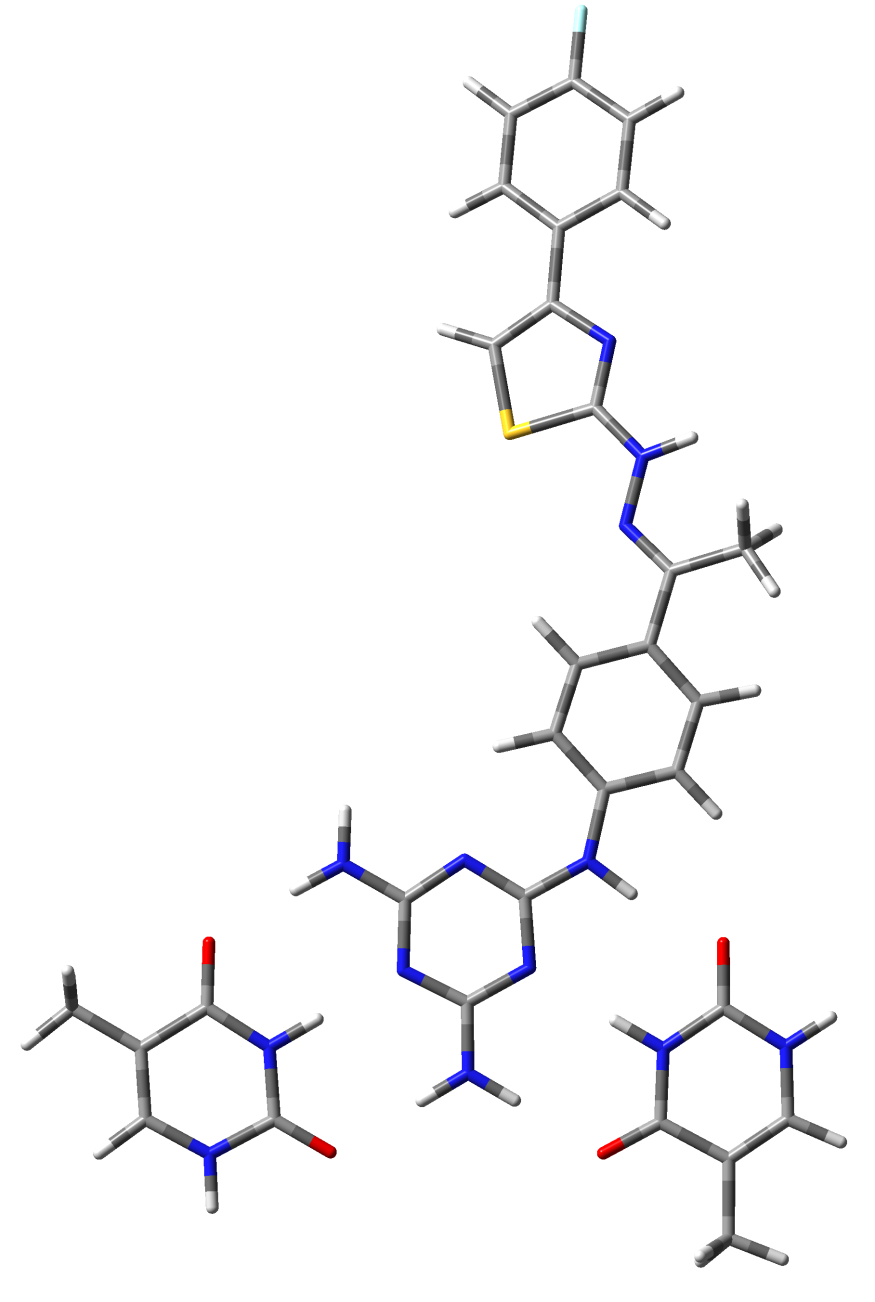
**

**(4a-T_2_)-II**

**
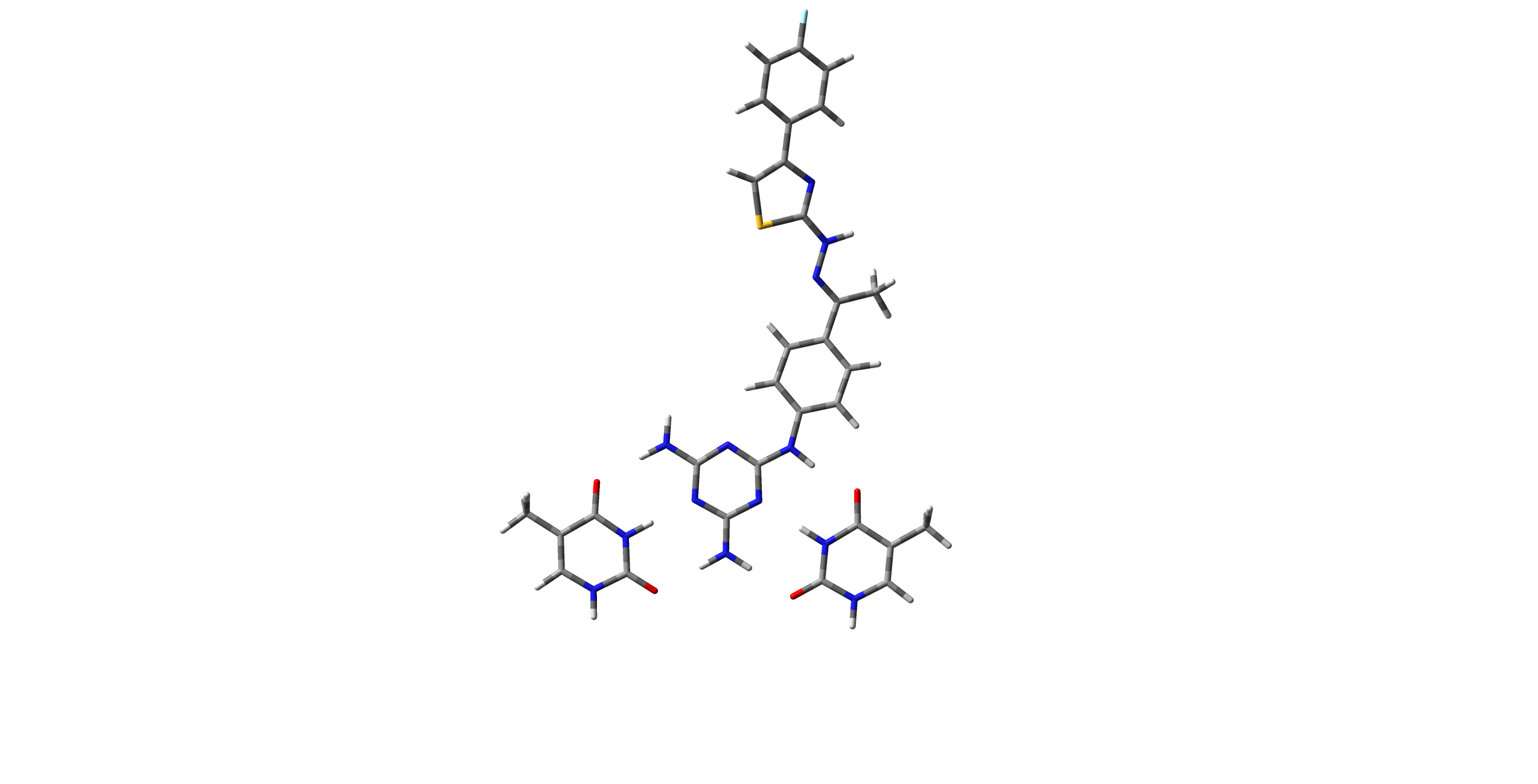
**

**(4a-T_2_)-III**

**
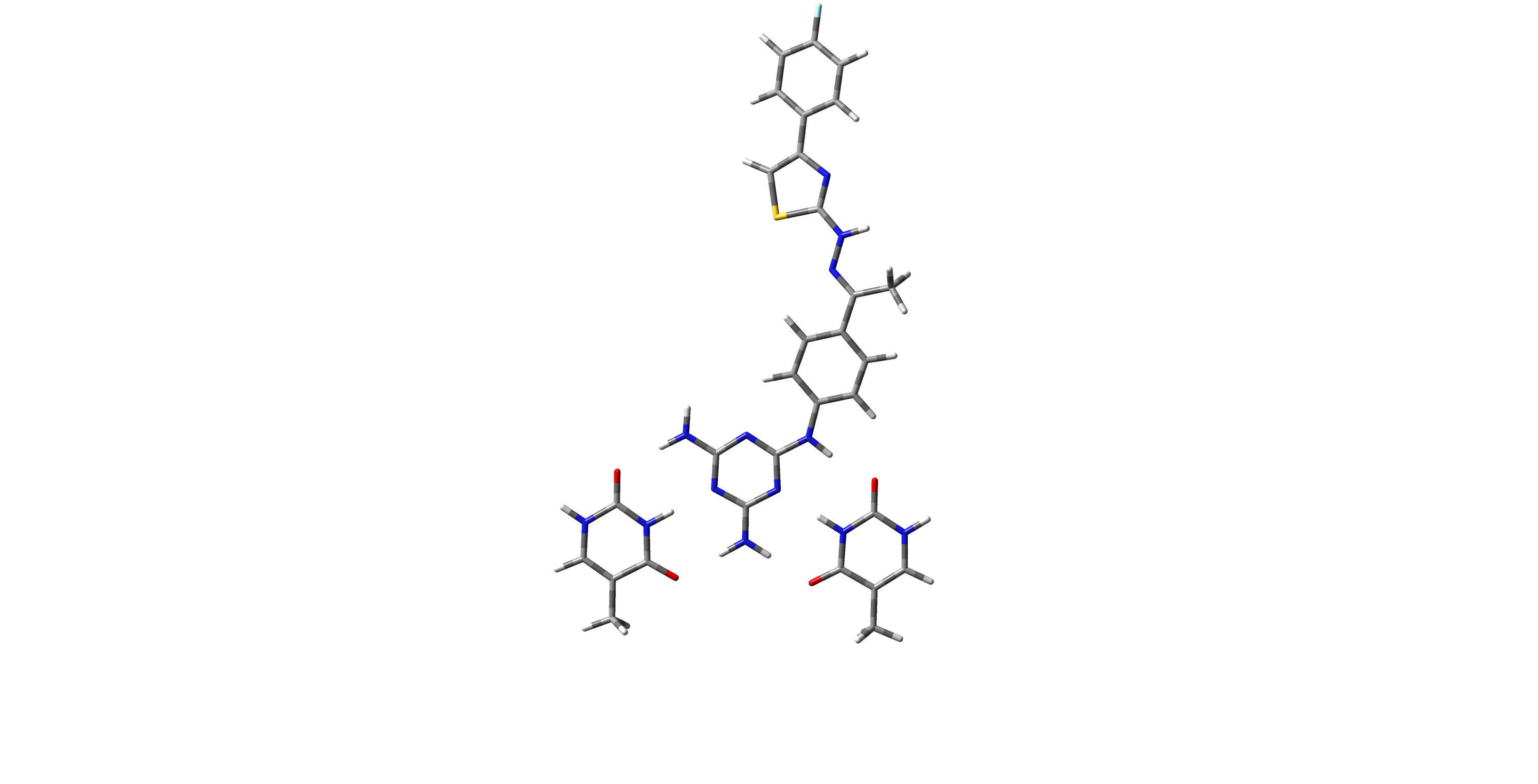
**

**(4a-T_2_)-IV
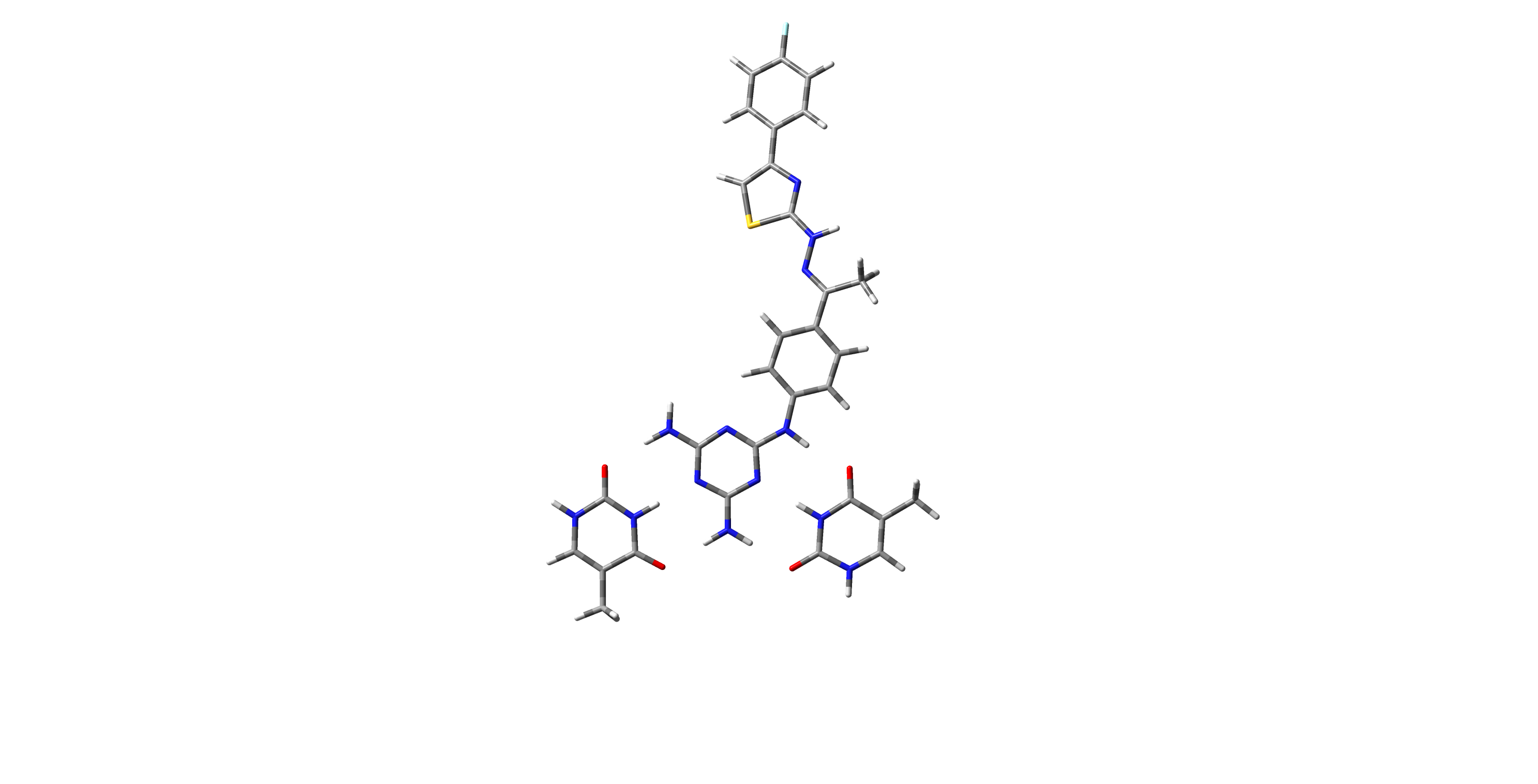
**

**S3.**

**(4a-T_2_)-I**

N 6.275096 3.773675 -0.028121

C 7.607251 3.454160 0.090526

N 8.448873 4.555015 0.115253

C 8.002821 5.854828 0.029574

C 6.694174 6.160747 -0.086629

C 5.734211 5.054201 -0.120621

O 4.523285 5.217335 -0.223559

O 8.036705 2.314695 0.168727

C 6.164414 7.560253 -0.182728

N 5.080137 -3.563087 0.190144

C 4.135237 -4.558176 0.162334

N 4.654873 -5.835626 0.266009

C 6.002368 -6.099347 0.386517

C 6.927481 -5.118725 0.412756

C 6.460479 -3.732924 0.308538

O 7.209503 -2.765328 0.321625

O 2.934313 -4.355445 0.055751

C 8.401961 -5.358003 0.542157

C 3.108830 1.699526 -0.199246

N 4.436572 1.518558 -0.086008

C 4.834436 0.227676 -0.000092

N 3.993653 -0.831329 -0.021911

C 2.682118 -0.517724 -0.139603

N 2.190226 0.715450 -0.231168

N 6.146722 -0.006772 0.113478

N 1.835509 -1.591637 -0.161534

N 2.646802 2.957857 -0.289365

C 0.439805 -1.672232 -0.268564

C -0.115072 -2.963287 -0.250622

C -1.483961 -3.151021 -0.360644

C -2.364919 -2.065606 -0.485555

C -1.797437 -0.781084 -0.497213

C -0.430824 -0.576212 -0.394764

C -3.827250 -2.248793 -0.580166

N -4.569076 -1.227135 -0.305553

N -5.915007 -1.344872 -0.397382

C -6.719550 -0.284666 -0.080501

S -6.043225 1.259974 0.413398

C -7.682481 1.843404 0.558805

C -8.581759 0.865294 0.240911

N -8.015321 -0.346076 -0.123802

C -4.415886 -3.577225 -0.997622

C -10.051706 0.985141 0.247589

C -10.699607 2.162028 0.652661

C -12.085485 2.259188 0.649628

C -12.831435 1.164319 0.237652

C -12.230597 -0.016255 -0.167317

C -10.842077 -0.099411 -0.159805

F -14.182151 1.254389 0.233714

H 5.610383 2.965186 -0.049747

H 9.431275 4.344214 0.201881

H 8.776339 6.612436 0.062100

H 3.269022 3.761735 -0.269185

H 6.800161 0.769210 0.133019

H 5.467640 7.768249 0.633652

H 6.973564 8.292416 -0.146511

H 5.606619 7.701001 -1.112396

H 4.713924 -2.587638 0.114864

H 3.976629 -6.581756 0.248145

H 6.263049 -7.148138 0.458317

H 6.504074 -0.954361 0.182814

H 2.299222 -2.494043 -0.083994

H 8.798938 -4.858199 1.429688

H 8.624801 -6.424495 0.611911

H 8.938314 -4.943198 -0.315385

H 1.653045 3.084593 -0.368707

H 0.540907 -3.819311 -0.146987

H -1.861795 -4.165649 -0.321887

H -2.453175 0.074234 -0.598281

H -0.020313 0.419423 -0.416050

H -6.384208 -2.213891 -0.623615

H -7.860732 2.861183 0.863708

H -5.113416 -3.439023 -1.831894

H -3.653554 -4.275112 -1.333462

H -4.969853 -4.048473 -0.176927

H -10.120740 3.017468 0.979396

H -12.591486 3.164343 0.961678

H -12.846420 -0.850014 -0.481407

H -10.348354 -1.010129 -0.470901

**(4a-T_2_)-II**

N -6.435852 3.727682 -0.017986

C -7.763093 3.390622 0.105695

N -8.619491 4.479932 0.130261

C -8.191451 5.785545 0.039918

C -6.887476 6.108854 -0.081159

C -5.912677 5.015316 -0.115387

O -4.704492 5.194619 -0.222513

O -8.177055 2.245580 0.188223

C -6.376994 7.515131 -0.182471

N -5.119842 -3.598062 0.216229

C -4.169361 -4.614797 0.188888

C -4.682474 -5.981085 0.304669

C -6.016639 -6.141273 0.426288

N -6.886474 -5.074637 0.443506

C -6.481044 -3.753619 0.339527

O -7.279533 -2.831991 0.358336

O -2.978399 -4.344799 0.073142

C -3.241746 1.696382 -0.193201

N -4.566909 1.498005 -0.075816

C -4.946521 0.202125 0.014615

N -4.092508 -0.845469 -0.006794

C -2.784820 -0.515076 -0.129116

N -2.310436 0.724828 -0.225127

N -6.256157 -0.048423 0.132539

N -1.924843 -1.577244 -0.150817

N -2.797086 2.960659 -0.287797

C -0.528166 -1.638741 -0.260893

C 0.044135 -2.922157 -0.241459

C 1.415215 -3.091326 -0.353989

C 2.281080 -1.994269 -0.482964

C 1.696293 -0.717594 -0.496127

C 0.327180 -0.531216 -0.391254

C 3.745532 -2.157962 -0.580194

N 4.474265 -1.126074 -0.308738

N 5.821589 -1.226372 -0.403195

C 6.612700 -0.155633 -0.088405

S 5.916847 1.380802 0.404244

C 7.548548 1.985643 0.547354

C 8.460111 1.018815 0.229957

N 7.909167 -0.200228 -0.132829

C 4.350912 -3.479178 -0.996635

C 9.928382 1.157761 0.235133

C 10.561461 2.341871 0.642584

C 11.945957 2.457111 0.637921

C 12.705535 1.373159 0.222016

C 12.119579 0.185903 -0.185213

C 10.732268 0.084635 -0.176070

F 14.054996 1.480838 0.216543

C -3.699939 -7.113678 0.281461

H -5.760075 2.928256 -0.039683

H -9.598656 4.256074 0.220543

H -8.975116 6.532624 0.072964

H -3.430009 3.756055 -0.268016

H -6.919431 0.719151 0.152459

H -5.824181 7.661017 -1.114318

H -7.195829 8.236398 -0.145400

H -5.680417 7.734533 0.631079

H -4.764770 -2.618575 0.136074

H -6.478958 -7.116599 0.517160

H -7.881282 -5.213308 0.535163

H -6.601087 -0.999094 0.206980

H -2.376388 -2.486738 -0.070421

H -1.805345 3.100554 -0.370294

H -0.599812 -3.786858 -0.134734

H 1.806763 -4.100676 -0.313902

H 2.340114 0.146363 -0.600369

H -0.096658 0.458765 -0.413843

H 6.301599 -2.089992 -0.627415

H 7.713921 3.006165 0.850344

H 4.912589 -3.941728 -0.176231

H 3.597204 -4.187624 -1.329777

H 5.044952 -3.333093 -1.832469

H 9.971923 3.188646 0.972798

H 12.440557 3.367895 0.951871

H 12.745778 -0.638957 -0.502275

H 10.250006 -0.831657 -0.488776

H -3.125509 -7.110493 -0.648712

H -2.977640 -7.017978 1.096516

H -4.205847 -8.076564 0.375394

**(4a-T_2_)-III**

N 6.197637 3.842232 -0.069043

C 7.556826 3.549969 0.047556

C 8.460972 4.703847 0.065944

C 7.914627 5.933071 -0.029643

N 6.555159 6.126420 -0.141922

C 5.635483 5.091396 -0.166767

O 4.435930 5.302342 -0.267662

O 7.941365 2.390298 0.127863

N 4.995759 -3.479660 0.191327

C 4.052348 -4.476208 0.171499

N 4.574107 -5.752228 0.281110

C 5.922389 -6.013275 0.399806

C 6.846101 -5.031196 0.418229

C 6.376789 -3.646640 0.307289

O 7.124124 -2.677844 0.313006

O 2.850772 -4.275803 0.066737

C 8.321260 -5.267426 0.545368

C 3.015568 1.777401 -0.223058

N 4.343314 1.600041 -0.111893

C 4.744321 0.309916 -0.019595

N 3.904773 -0.750430 -0.033572

C 2.592718 -0.439916 -0.150083

N 2.098334 0.792099 -0.247483

N 6.056804 0.079654 0.092186

N 1.747742 -1.515270 -0.164127

N 2.549507 3.034628 -0.319385

C 0.352017 -1.598778 -0.267987

C -0.200750 -2.890599 -0.240843

C -1.569516 -3.081237 -0.347241

C -2.452487 -1.998075 -0.477492

C -1.887123 -0.712742 -0.498325

C -0.520675 -0.505019 -0.399611

C -3.914671 -2.184189 -0.568423

N -4.657674 -1.161990 -0.299015

N -6.003594 -1.282455 -0.387943

C -6.809284 -0.221564 -0.076488

S -6.134544 1.326980 0.407393

C -7.774470 1.908944 0.551379

C -8.672731 0.927665 0.240458

N -8.105023 -0.285034 -0.117784

C -4.501862 -3.516159 -0.976521

C -10.142838 1.045487 0.248177

C -10.791941 2.223416 0.648257

C -12.177954 2.318661 0.646178

C -12.922817 1.220789 0.240265

C -12.320769 0.039082 -0.159575

C -10.932130 -0.042128 -0.153099

F -14.273648 1.308994 0.237286

C 9.932787 4.445905 0.189852

H 5.535447 3.031547 -0.084106

H 8.511041 6.837253 -0.023704

H 6.163408 7.052986 -0.212416

H 3.168320 3.839789 -0.304298

H 6.708975 0.858161 0.105143

H 4.628138 -2.505247 0.111427

H 3.896977 -6.499471 0.269142

H 6.184768 -7.061265 0.476896

H 6.416316 -0.866798 0.165795

H 2.213110 -2.416511 -0.082289

H 8.719583 -4.761951 1.429069

H 8.545983 -6.333155 0.620560

H 8.854840 -4.856516 -0.315762

H 1.555219 3.158228 -0.396334

H 0.456834 -3.744863 -0.132974

H -1.945606 -4.096217 -0.301375

H -2.544461 0.140829 -0.603709

H -0.111821 0.491114 -0.427964

H -6.471749 -2.153531 -0.608358

H -7.953834 2.928300 0.850302

H -5.200882 -3.384365 -1.810583

H -3.738952 -4.214969 -1.309096

H -5.053823 -3.983032 -0.151987

H -10.213915 3.081174 0.970376

H -12.684893 3.224595 0.954422

H -12.935763 -0.797044 -0.468957

H -10.437477 -0.953678 -0.460253

H 10.499143 5.379370 0.191515

H 10.154355 3.900057 1.110816

H 10.287682 3.822210 -0.634919

**(4a-T_2_)-IV**

N -6.354932 -3.804597 -0.059820

C -7.710005 -3.495849 0.060555

C -8.628397 -4.638355 0.078876

C -8.097637 -5.874143 -0.020225

N -6.741039 -6.084154 -0.135993

C -5.808567 -5.060569 -0.161142

O -4.612070 -5.286253 -0.265244

O -8.080146 -2.331580 0.144042

N -5.040843 3.507553 0.214569

C -4.093013 4.526790 0.196371

C -4.610127 5.891020 0.317962

C -5.945188 6.047013 0.435615

N -6.812289 4.978137 0.443687

C -6.403024 3.658701 0.333809

O -7.198864 2.734858 0.344567

O -2.900853 4.260506 0.083396

C -3.147368 -1.779270 -0.217313

N -4.472874 -1.586011 -0.102720

C -4.857193 -0.291276 -0.006552

N -4.005591 0.758645 -0.020005

C -2.696897 0.432830 -0.140385

N -2.218524 -0.805408 -0.241634

N -6.167421 -0.046403 0.108806

N -1.839750 1.497438 -0.154053

N -2.697155 -3.041989 -0.317507

C -0.443051 1.563459 -0.260583

C 0.125685 2.848300 -0.231602

C 1.496508 3.021964 -0.340249

C 2.365685 1.928169 -0.474638

C 1.784461 0.650006 -0.497299

C 0.415668 0.459188 -0.396418

C 3.829854 2.096490 -0.567971

N 4.560904 1.064833 -0.301869

N 5.908148 1.169424 -0.393258

C 6.701590 0.098813 -0.084119

S 6.008992 -1.442374 0.398313

C 7.642058 -2.043957 0.539860

C 8.551558 -1.072895 0.229560

N 7.998000 0.146916 -0.126574

C 4.432386 3.422026 -0.974684

C 10.020165 -1.208211 0.235651

C 10.655766 -2.392460 0.638739

C 12.040542 -2.504305 0.634835

C 12.797842 -1.416760 0.224183

C 12.209353 -0.229209 -0.178514

C 10.821793 -0.131368 -0.170209

F 14.147555 -1.521129 0.219480

C -3.630567 7.026358 0.304678

C -10.096631 -4.362473 0.206649

H -5.682502 -3.002204 -0.074962

H -8.705281 -6.770821 -0.014555

H -6.360968 -7.015382 -0.209102

H -3.325747 -3.839453 -0.302424

H -6.828625 -0.817255 0.121735

H -4.683118 2.529508 0.130251

H -6.410343 7.020635 0.530261

H -7.807800 5.113666 0.532526

H -6.515714 0.902783 0.186577

H -2.294032 2.405258 -0.069481

H -1.704612 -3.177646 -0.396749

H -0.520893 3.710491 -0.120599

H 1.885159 4.032112 -0.292797

H 2.430909 -0.211451 -0.605920

H -0.005388 -0.531780 -0.426306

H 6.386172 2.035658 -0.611556

H 7.809671 -3.065949 0.836601

H 5.128203 3.283320 -1.810301

H 3.677344 4.130635 -1.304424

H 4.991447 3.880638 -0.150298

H 10.067996 -3.241978 0.965040

H 12.537098 -3.415161 0.945473

H 12.833819 0.598524 -0.491478

H 10.337579 0.785082 -0.479419

H -2.910988 6.928081 1.121836

H -3.052743 7.029961 -0.623394

H -4.139430 7.987338 0.402133

H -10.674434 -5.288893 0.208090

H -10.309350 -3.815546 1.129063

H -10.445767 -3.733128 -0.616296
